# Comparative transcriptome analysis of canola carrying a single vs stacked resistance genes against clubroot

**DOI:** 10.3389/fpls.2024.1358605

**Published:** 2024-05-21

**Authors:** Rui Wen, Tao Song, Bruce D. Gossen, Gary Peng

**Affiliations:** Saskatoon Research and Development Centre, Agriculture and Agri-Food Canada, Saskatoon SK, Canada

**Keywords:** *Brassica napus*, *Plasmodiophora brassicae*, pathotype, resistance pyramiding, RNA-seq, PAMPS, PTI and ETI

## Abstract

Pyramiding resistance genes may expand the efficacy and scope of a canola variety against clubroot (*Plasmodiophora brassicae*), a serious threat to canola production in western Canada. However, the mechanism(s) of multigenic resistance, especially the potential interaction among clubroot resistance (CR) genes, are not well understood. In this study, transcriptome was compared over three canola (*Brassica napus* L.) inbred/hybrid lines carrying a single CR gene in chromosome A03 (*CRa^M^*, Line 16) or A08 (*Crr1^rutb^*, Line 20), and both genes (*CRa^M^* +*Crr1^rutb^*, Line 15) inoculated with a field population (L-G2) of *P. brassicae* pathotype X, a new variant found in western Canada recently. The line16 was susceptible, while lines 15 and 20 were partially resistant. Functional annotation identified differential expression of genes (DEGs) involved in biosynthetic processes responsive to stress and regulation of cellular process; The Venn diagram showed that the partially resistant lines 15 and 20 shared 1,896 differentially expressed genes relative to the susceptible line 16, and many of these DEGs are involved in defense responses, activation of innate immunity, hormone biosynthesis and programmed cell death. The transcription of genes involved in Pathogen-Associated Molecular Pattern (PAMP)-Triggered and Effector-Triggered Immunity (PTI and ETI) was particularly up-regulated, and the transcription level was higher in line 15 (*CRa^M^* + *Crr1^rutb^*) than in line 20 (*Crr1^rutb^* only) for most of the DEGs. These results indicated that the partial resistance to the pathotype X was likely conferred by the CR gene *Crr1^rutb^* for both lines 15 and 20 that functioned via the activation of both PTI and ETI signaling pathways. Additionally, these two CR genes might have synergistic effects against the pathotype X, based on the higher transcription levels of defense-related DEGs expressed by inoculated line 15, highlighting the benefit of gene stacking for improved canola resistance as opposed to a single CR gene alone.

## Introduction

Clubroot, caused by the soil-borne protist *Plasmodiophora brassicae* Woronin, is an important disease of brassica crops worldwide (Dixon, 2009), and a serious threat to canola production in western Canada where the crop contributes billions to the annual economy (Rempel et al., 2014; Strelkov and Hwang, 2014; Gossen et al., 2015). In the realm of managing the disease on canola, genetic resistance has proven to be both efficacious and practical, particularly when used with extended crop rotations exceeding a 2-year break from a canola crop that drastically diminishes *P. brassicae* inoculum in the soil (Peng et al., 2014; Peng et al., 2015). Despite the effectiveness, repeated uses of a single clubroot resistance (CR) gene has led to rapid resistance erosion in Canada, a consequence of the heightened selection pressure from a diverse pathogen population (Strelkov et al., 2018; Mcdonald et al., 2020). Novel *P. brassicae* pathotypes have been identified since the introduction of resistant canola cultivars in 2009, including pathotype X. These ‘new’ pathotypes defeated the single CR genes used in earlier resistant canola cultivars released in Canada (first-generation) within 3-4 years (Strelkov et al., 2018; Sedaghatkish et al., 2019).

It has been recognized that CR resources are limited (Hirai, 2006). To date 18 major CR loci have been reported, with most of them being originated from European turnip (*B. rapa*). Many of these CR loci belong to the toll-interleukin receptor (TIR)-nucleotide-binding site (NBS) family (Karim et al., 2020; Mehraj et al., 2020). Two cloned CR genes, *CRa* and *Crr1*, also encode TIR-NBS-LRR (leucine-rich repeat) proteins (Ueno et al., 2012; Hatakeyama et al., 2013). In Canada, one of the CR genes derived from the winter rapeseed cultivar ‘Mendel’ (A03) appeared to be present in most first-generation resistant canola cultivars post-2009 (Fredua-Agyeman and Rahman, 2016). While effective against initial five pathotypes found in Canada, this gene was observed to have lost the effectiveness in some field by 2013 (Strelkov et al., 2018; Karim et al., 2020). Additional CR genes (A08) derived from rutabaga (Hasan and Rahman, 2016) were introduced later, which showed resistance or moderate resistance to most of the novel pathotypes, including pathotype X (Tonu et al., 2023). The rutabaga-derived CR gene(s) have also been pyramided with those on A03 (second-generation CR) for a broader range of efficacy.

While stacking resistance genes has proven effective against multiple pathogen races in other crops, including rice against bacterial blight (Li et al., 2001), potentially prolonging the useful life of individual genes, the situation in canola/rapeseed, especially concerning resistance to blackleg (*Leptosphaeria maculans*), appears complex (Balesdent et al., 2022). In comparison, combining major *R* genes with quantitative resistance consistently enhanced the resistance durability (Brun et al., 2010). Stacking three CR genes in Chinese cabbage (*B. rapa*) expanded the resistance against multiple *P. brassicae* pathotypes (Matsumoto et al., 2012), although the resistance mechanisms associated with CR-gene stacking remain unexplored. In a recent study, Tonu et al. (2023) reported that canola varieties carrying stacked CR genes (A03 and A08) had greater resistance durability than those carrying either CR gene alone, when exposed repeatedly to a field *P. brassicae* pathotype X population. It was unclear, however, whether these stacked CR genes would provide more sophisticated resistance mechanisms than a single CR gene alone against novel pathotypes virulent towards the first-generation resistant canola cultivars.

Studying molecular mechanisms, particularly those conferred by both single and stacked CR genes, may help understand the basis of resistance for developing genetic resources efficiently. As single and stacked genes provide resistance against particular pathotypes/strains of the pathogen, understanding the modes of action may allow for a better design in CR breeding, enabling the development of canola cultivars with tailored resistance profiles. This type of study may also help identify gene combinations that confer more durable resistance, aiding in judicious deployment of genetic resources. For instance, breeders can use complementary CR genes which offer the best protection against prevailing pathogen populations based on the understanding of synergistic or additive effects of gene stacking.

Transcriptome analysis, aimed at identifying differentially expressed genes (DEGs) between susceptible and resistant plants, serves as a powerful tool for unraveling the biological pathways in the host against pathogen attacks (Orshinsky et al., 2012; Soneson and Delorenzi, 2013). The comparative analysis can also shed light on defense pathways triggered by specific CR genes or their combinations. The primary objective of this study was to the novel pathotype X as a model to unravel the molecular mechanisms conferred by double (A03 and A08) vs. single (A03 or A08) CR genes, probing for potential interactions in mediating the resistance. This was achieved through: 1) Identifying DEGs between canola lines carrying single and double CR genes inoculated with the pathotype X; 2) conducting functional analysis of DEGs to identify biological pathways crucial for the resistance; and 3) determining distinct defense mechanisms and transcription levels activated by specific CR genes or gene combinations. The information holds practical value for designing CR-gene stacking in canola breeding and for aiding deployment of cultivars equipped with optimized CR genes for enhanced resistance performance and durability.

## Materials and methods

### Plant materials, inoculation and disease assessment

Three commercial canola inbreed/hybrid lines/varieties, designated as line 15, 16 and 20 in this study, were provided by Nutrien Ag Solutions, Saskatoon, Saskatchewan, Canada. Line 16 carries a single CR gene in Chromosome A03 that was derived from the winter oilseed rape cultivar Mendel. This CR gene was originally from a fodder turnip (*B. rapa*) used in the European Clubroot Differentials (ECD) designated as ECD4. This ECD differential was believed to carry three CR genes but two of them might have been lost during backcrossing with *B. napus* in production of the oilseed rape cultivar Mendel (Diederichsen et al., 2006). Fredua-Agyeman and Rahman (2016) showed that this CR locus was physically close to that of *CRa*/*CRb* ^Kato^ located in Chromosome A03 of *B. rapa* (Matsumoto et al., 1998; Kato et al., 2012; Ueno et al., 2012; Kato et al., 2013). Recent evidence showed that this CR gene from ‘Mendel’ is identical to *CRa* (Hu et al., 2024).

Line 20 carries CR gene(s) in Chromosome A08 derived from a variety of rutabaga (*B. napus* var. *napobrassica*); Composite Interval Mapping analysis showed that this CR locus can be flanked by the SSR markers sS1702 and A08_5024 (Hasan and Rahman, 2016), which would indicate a region in A08 where the CR gene *Crr1* is also located (Suwabe et al., 2012; Hatakeyama et al., 2013). Based on fine mapping of a 1.6 cM region on A08, Suwabe et al. (2012) provided the evidence that the *Crr1* region would carry two CR genes, i.e., *Crr1a* and *Crr1b* in a very close range. However, it is unclear whether line 20 carries only a single or both alleles in A08. To avoid nomenclature confusion, the CR gene(s) carried in lines 16 and 20 were respectively referred to as *CRa^M^* and *Crr1^rutb^* in this paper to reflect their origins and relatedness to *CRa* and *Crr1*.

Line 15, on the other hand, was produced by the hybridization of inbred lines 16 and 20, which would carry at least two dominant CR genes derived from A03 and A08, respectively, based on marker analysis (data not shown). However, it remains undetermined whether a single or two CR genes were present in A08 of the hybrid. These inbred/hybrid lines (15, 16 and 20) were used to assess and compare the transcriptomic responses of *CRa^M^* and *Crr1^rutb^*, both individually and in combination.

A field collection (L-G2) of *P. brassicae* pathotype X was used throughout the study to inoculate all canola lines. Pathotype X was the first strain characterized to be virulent on the first generation of resistant canola cultivars in Canada (Strelkov et al., 2016). The clubroot reaction of the three lines to L-G2 was compared against a mock treatment as a negative control and the susceptible canola cultivar Westar as a positive control.

A resting spore suspension of L-G2 was prepared and mixed into soil-less Sunshine #3 potting mix (pH = 6.2. SunGro Horticulture, Vancouver, BC) to reach 1 x 10^6^ spores/g soil concentration. Subsequently, each line was planted in infested growth medium (20 cm in diameter, 15 cm deep) with 20 seeds per pot. The pots were placed in a growth room set at 22°C/16°C (day/night) with a 16 h photoperiod until root sampling. Following seeding, the growth medium in each pot was initially saturated for one week and then maintained in a moist condition through regular watering.

Root tissues were collected from each plant at 14 days post inoculation (dpi), with 15 plants per cultivar per pot (replicate) and three replicates in total for each line. The soil was rinsed off from the roots with tap water to preserve the whole root system, and then the entire root system was cut, flash frozen in liquid nitrogen and stored at -80°C until use. Five remaining plants from each pot were maintained in the growth room and assessed for clubroot severity on a standard 0–3 scale at 35 dpi to confirm the success of inoculation. A disease severity index (DSI) was calculated for the five remaining plants assessed for each replicate (Tonu et al., 2023).

### RNA-seq analysis

RNA extraction from root samples was performed using the RNeasy Plant Mini Kit (Qiagen, Toronto, ON) following the manufacturer’s instructions, with DNase digestion using the RNase-Free DNase Set from Qiagen. The quality and concentration of RNA were evaluated using the Experion RNA StdSens Analysis Kit on the Experion automated electrophoresis system (Bio-Rad, Montreal, QC) and Nanodrop 2000c (Fisher Scientific, Toronto, ON) to ensure sufficient RNA quality and quantity for cDNA library preparation.

cDNA libraries were prepared using the TruSeq RNA Sample Preparation Kit v2 (Illumina; San Diego, CA). The quality and concentration of cDNA were assessed also with the Experion DNA Analysis Kit and Nanodrop 2000c. These libraries were sent to the Génome Québec Innovation Centre, McGill University for RNA sequencing on the Illumina Hiseq 2500 platform. The raw data of RNA-seq has been deposited at the National Center for Biotechnology Information (NCBI) under this ID number: SUB14143825.

RNA-seq data processing and DEG analysis followed the protocol outlined by Chu et al. (2014). Briefly, CLC Genomics Workbench v10.1.1 (Qiagen) was used to process and analyze raw sequencing data (FASTQ files); the reads underwent quality control checks, followed by trimming to remove Illumina adapters and low-quality reads. Clean reads were aligned to the reference genome of *Brassica napus* (v4.1; http://brassicadb.org/brad). The level of gene expression was quantified in Reads Per Kilobase of exon model per Million reads (RPKM). There are concerns about incorrect uses of RPKM for some samples (Evans et al., 2018; Zhao et al., 2020), and we have been keeping all plants grown under the same environment and using the same RNA-seq protocol on all samples. To minimize unnecessary data analyses due to excessively intricate interactions, DEGs were identified at RPKM |>4| to better focus on most highly differentially-expressed genes relevant to CR functions. Such strategy has been used in other RNA-seq data analyses (Rosli et al., 2013; Chhiba et al., 2017; Iqbal & Kumar, 2022; Jayavelu et al., 2023). A false discovery rate (FDR) threshold was set at *P* ≤ 0.01. DEG comparisons were made between the inoculated treatment and the negative control (mock) for each canola variety/line.

### Annotation of DEGs

The list of DEGs underwent gene ontology (GO) annotation using Blast2GO Pro (Conesa et al., 2005) with BLASTx algorithms against the non-redundant protein database provided by the National Center for Biotechnology Information (http://www.ncbi.nlm.nih.gov). DEGs were classified into three GO classes: biological process, molecular function and cellular component. The enrichment analysis was conducted by using a built-in tool, Fisher’s Exact Test, in Blast2GO Pro. Heatmaps and gene clustering were performed using a R package (https://CRAN.Rproject.org-/package=pheatmap), and Log-transformation was applied prior to plotting. The annotations were further analyzed using MapMan software (Thimm et al., 2004) to categorize abiotic and biotic pathways associated with selected DEGs.

### Verification of RNA-seq data quality

To ensure the reliability of RNA-seq data, droplet digital PCR (ddPCR) was conducted using a QX200™ System (Bio-Rad). Reagents and consumables were also from the same supplier, including droplet generator oil, DG8TM cartridges and gaskets, droplet reader oil and ddPCR EvaGreen supermix. For each sample, 20-µl reaction mix containing 10-µl EvaGreen supermix, 1 µl each of forward and reverse primers (22.5 µM), 2-µl sample complementary DNA and 6-µl double distilled H_2_O was partitioned into aqueous droplets in oil via a droplet generator. The droplet suspension was transferred to a 96-well PCR plate, and a thermocycling process carried out in a conventional thermal cycler (Thermo Fisher Scientific Canada, Toronto, ON) at 95°C for 5 min, 95°C for 30 s (40 cycles), 56-62°C (depending on specific genes) at ramp rate: 2°C/s for 60 s (40 cycles), 4°C for 5 min, 90°C for 5min and then, 4°C infinite. Then the PCR plate was determined for the fraction of PCR-positive droplets in each sample on a droplet reader, and the data analyzed using the QuantaSoft™ Software. The expression patterns of 10 selected DEGs, involved in different biological process, were analyzed using droplet digital PCR (ddPCR). The primers for amplifying these genes were listed in **Supplementary Table 1**.

### Statistical analysis

DSI data were transformed (arcsine square root) and normal distribution confirmed based on Shapiro–Wilk Test (PROC UNIVARIATE) using SAS v9.3 (SAS Institute, Cary, NC, USA) prior to statistical analyses. The homogeneity of variance was assessed using Levene’s Test. Fisher’s Protected LSD was used to separate treatment means when ANOVA was significant (*P* ≤ 0.05).

## Results

### Clubroot severity on inbred and hybrid canola lines

**Figure 1:**
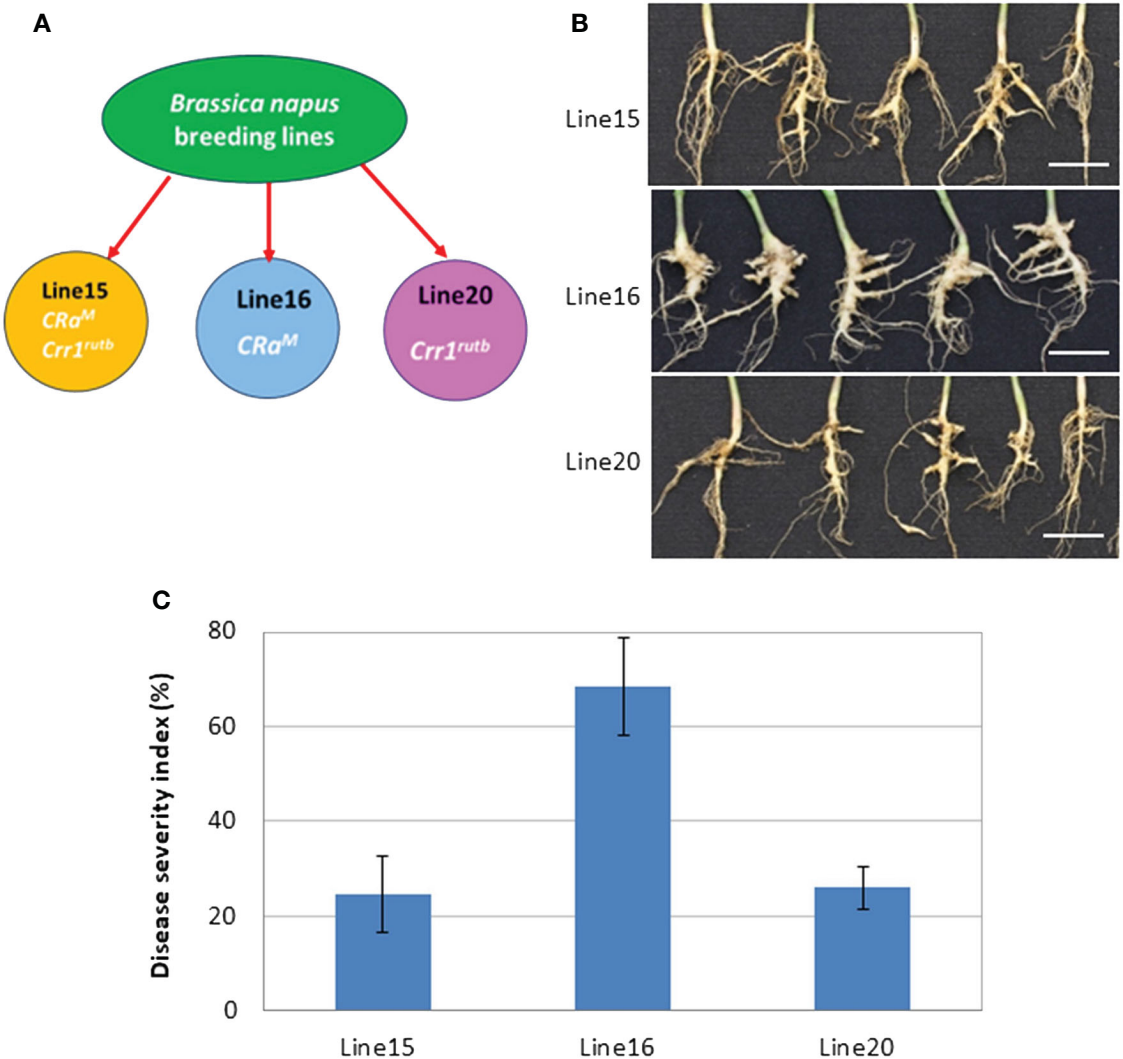
The mean disease severity index (DSI) of three canola lines carrying *CRa^M^* (line 16), *Crr1^rutb^* (line 20) and *CRa^M^* + *Crr1^rutb^* (line 15), in response to inoculation with L-G2 of *Plasmodiophora brassicae* pathotype X: **(A)** schematic diagram illustrating the lines with different resistance genes; **(B)** Symptoms at 35 days after inoculation (dpi), scale bar = 1cm; **(C)** DSI (n=6).

In this investigation, we evaluated the impact of CR genes *CRa^M^* and *Crr1* ^rutb^, individually and in combination, against the L-G2 collection of pathotype X. Line 16, carrying the single CR gene *CRa^M^*, was susceptible with severe clubroot symptoms visible at 35 dpi. In contrast, lines 20 and 15, carrying *Crr1^rutb^* and both *CRa^M^* and *Crr1^rutb^*, respectively, displayed partial resistance with only slight to moderate symptoms (**Figures 1A, B**). The disease severity indices (DSIs) were 68% for line 16, 26% for line 20, and 24% for line 15 (**Figure 1C**).

### RNA-seq analysis

RNA-seq analysis was conducted on root samples from the three *B. napus* lines carrying *CRa^M^*, *Crr1^rutb^*, or both CR genes collected at 14 dpi with three biological replicates. A total of 1.08 billion reads were generated through 100 bp paired-end sequencing from 18 cDNA libraries (**Supplementary Table 2**). Approximately one-third of the total reads were obtained from each line: 368,201,810 from line 15 (166,577,030 for control and 201,624,780 for inoculated, respectively), 337,590,744 from line 16 (175,260,710 for control and 162,330,034 for inoculated), and 377,931,694 from line 20 (189,199,162 for control and 188,732,532 for inoculated).

The GC content ranged from 46-48%, and all libraries contained >80% of sequences with an average PHRED score >30 (Q30%), ensuring the quality and accuracy of the sequencing data for further analyses and gene expression comparisons. Sequences were trimmed to remove impurities (i.e., low-quality reads, adapters etc.) before the analysis of RNA-seq data. Around 70% of paired-sequence reads for each cDNA library were mapped to the *B. napus* reference genome version 4.1 (http://brassicadb.org). A summary of RNA-seq data quality checks and analyses is presented in **Supplementary Table 2**.

**Figure 2:**
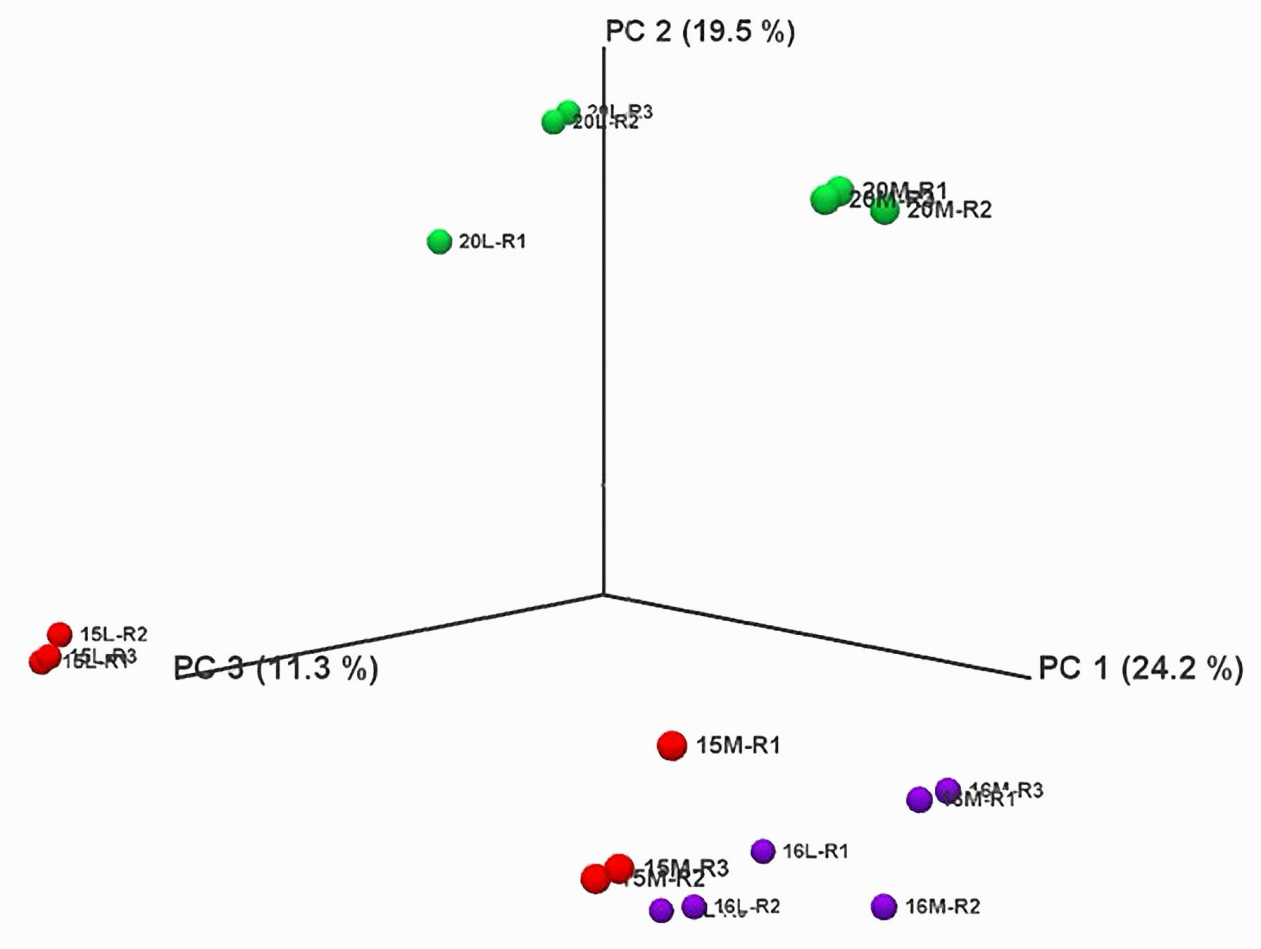
Principal component analysis of RNA-Seq data over all replicates of root samples from three canola lines inoculated with L-G2 of *Plasmodiophora brassicae* pathotype X. Overlapping regions correspond to the number of DEGs identified in different biological replicates. Red marks represent line 15, purple marks for line 16, and green marks for line 20. M represents mock inoculation, and L represents L-G2 of pathotype X inoculation. Samples used for RNA-seq were collected at 14 dpi.

Principal Component Analysis (PCA) revealed that biological replicates were grouped together for each treatment in the same dimension, indicating no major variations in the RNA-seq data within each treatment (**Figure 2**; **Supplementary Figure 1**). Additionally, the three biological replicates of control and inoculated treatments were closely clustered for line 16, suggesting no major differences between the control and inoculated samples. Conversely, the replicates of control and inoculated treatments for lines 15 and 20 were in different dimensions, indicating significant differences in the RNA-seq data.

### Annotation of DEGs

**Figure 3:**
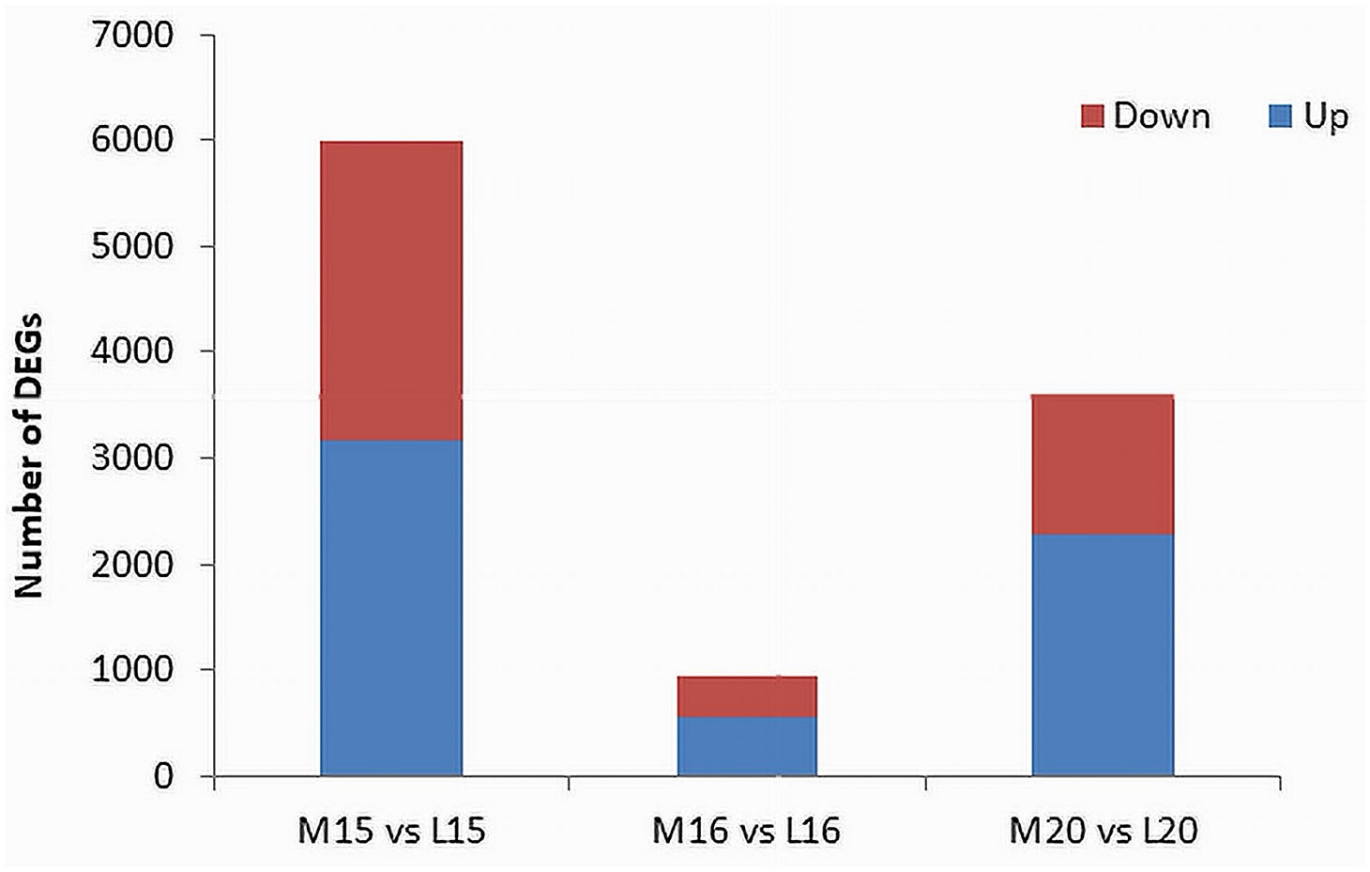
Identification of DEGs filtered by an absolute fold change > 4 and *P*-value ≤ 0.01 between inoculated and control root samples of three canola lines. The red portions at the top of each bar represent down-regulated genes in the inoculated plants relative to the control, and the blue portions at the bottom represent up-regulated genes.

The threshold for the identification of DEGs was set at an absolute fold change of ≥ 4 and a False Discovery Rate (FDR) *P*-value ≤ 0.01. In line 15, a total of 6,000 DEGs were identified, comprising 3,163 up-regulated genes and 2,837 down-regulated genes in the inoculated plants compared to the control. For line 16, 953 DEGs were identified, with 546 up-regulated and 407 down-regulated genes. Line 20 exhibited 3,613 DEGs, including 2,286 up-regulated and 1,327 down-regulated genes (**Figure 3**; **Supplementary Table 3**).

**Figure 4:**
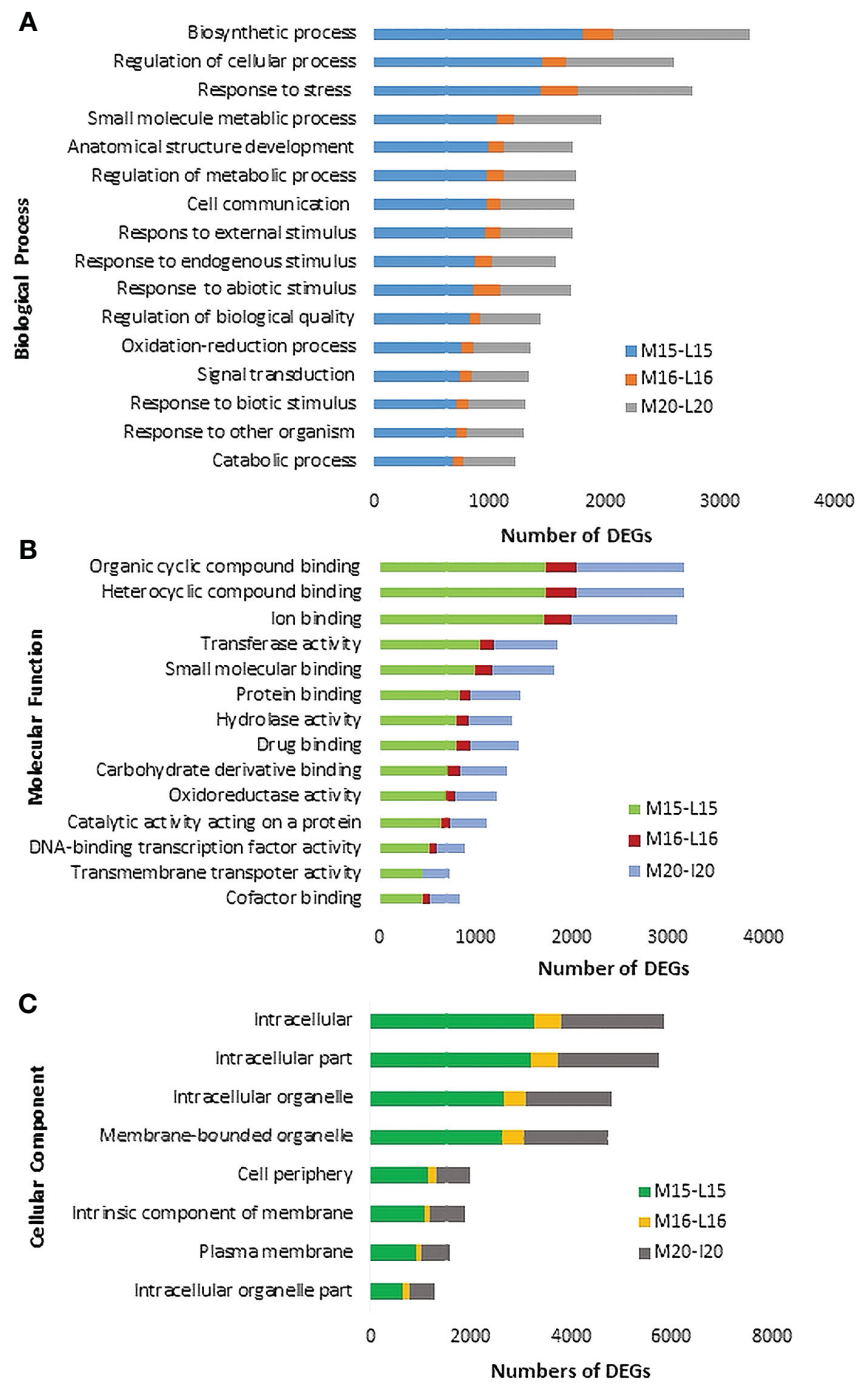
Gene ontology (GO) assignment of DEGs identified in selected Biological Processes **(A)**, Molecular Functions **(B)** and Cellular Components **(C)** of three canola lines.

**Figure 5:**
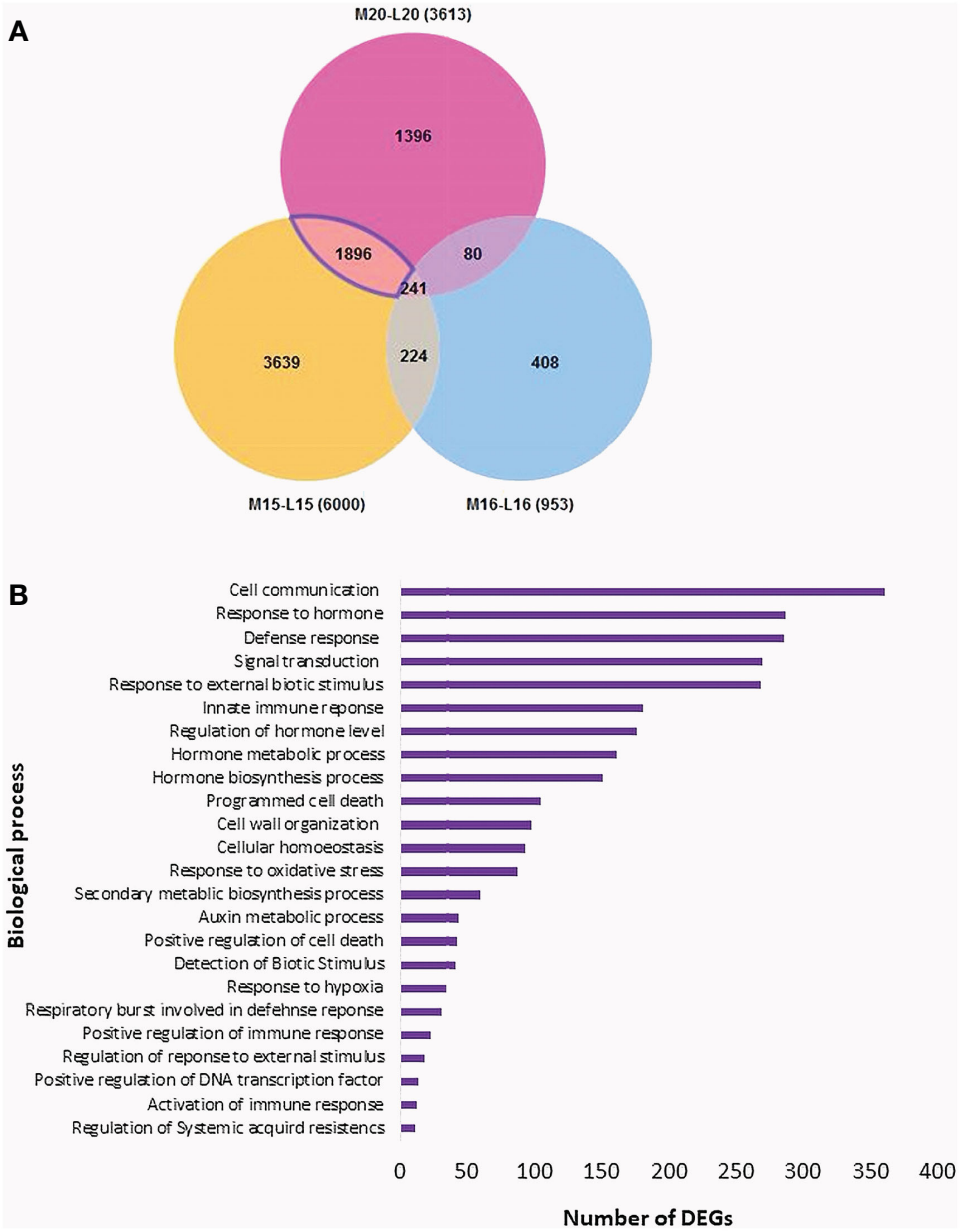
Distribution of DEGs and their biological process analysis for three canola lines: **(A)** Venn diagram of the distribution of DEGs; **(B)** Enrichment analysis of the DEGs in disease resistance-related biological processes shared by lines 15 and 20, but not 16.

The Blast2GO analysis revealed that up- and down-regulated DEGs were mainly involved in cellular processes, metabolic processes, response to stimuli, and biological regulation. The total number of up- and the DEGs across the three canola lines are shown in **Supplementary Figure 2**. GO analysis revealed DEGs associated with Biological Processes, Molecular Functions and Cellular Components; most DEGs from inoculated plants were involved in the biological processes of biosynthesis, response to stress and regulation of cellular processes (**Figure 4A**). Line 15 had the most DEGs assigned to each biological process compared to line 20 or line16. For molecular functions, a substantial proportion of DEGs were associated with organic cyclic compound binding, heterocyclic compound binding, ion binding and transcription factor activity (**Figure 4B**). Regarding cellular components, DEGs were relatively abundant in intracellular parts, followed by intracellular organelles and membrane-bounded organelles (**Figure 4C**). Furthermore, enrichment analysis was performed on identified DEGs using Fisher’s Exact Test in Blast2GO, with The top 20 enriched GO terms identified in **Supplementary Figure 3**. The highest number of DEGs were linked to cellular anatomical entities. Interestingly, DEGs associated with cellular processes ranked as the second most common in lines 15 and 20, but were not present in significant numbers in line 16. Lines 15 and 20 (partially resistant) appeared to share many common DEGs and functions in similar biological processes, while the susceptible line 16 lacked most of these DEGs against the infection by pathotype X; Venn diagram analysis showed that 1,896 DEGs were shared between lines 15 and 20 (**Figure 5A**; **Supplementary Table 4**). The enrichment analysis of these DEGs revealed their involvement in the biological process of cell communication and responses to hormones and defense responses, with some of the DEGs functioning in programmed cell death and the regulation of systemic acquired resistance (SAR) (**Figure 5B**).

Further analysis of DEGs involved in defense response categories, shared by lines 15 and 20 but not line 16, showed the activation of genes involved in signaling pathways PTI and ETI, respectively (**Tables 1**, **2**; **Supplementary Tables 5**, **6**). Additionally, PTI signaling pathways involved pattern recognition receptors (PRRs), mitogen-activated protein kinase (MAPK) cascade, and transcription factors (TF). Key PRRs included wall-associated receptor kinase-like 10, cysteine-rich receptor-like protein kinase 36, cysteine-rich receptor-like protein kinase 10 isoform x2 and receptor-like protein 12, with the most significant fold changes observed in 1ine 15 (**Table 1**; **Supplementary Figure 4A**).

**Table 1:** List of top 10 Pattern Recognition Receptors (PRRs) and transcription factors involved in Pattern-Triggered Immunity (PTI) based on fold changes in gene regulation associated with inoculation of three canola lines.

Gene ID	Gene name	15-M vs 15-I	16-M vs 16-I	20-M vs 20-I
BnaA07g20220D	wall-associated receptor kinase-like 10	1400.62	2.78	9.59
BnaA09g20420D	cysteine-rich receptor-like protein kinase 36	760.20	6.15	36.58
BnaCnng35600D	cysteine-rich receptor-like protein kinase 10 isoform x2	575.00	-1.34	26.50
BnaA08g09000D	receptor-like protein 12	331.8	4.88	11.78
BnaCnng25710D	cysteine-rich receptor-like protein kinase 36	90.66	2.48	11.72
BnaA06g12200D	receptor-like protein 12	68.59	3.4	10.03
BnaA03g45820D	cysteine-rich receptor-like protein kinase 15 isoform x1	60.35	7.53	36.29
BnaC05g27560D	probable lrr receptor-like serine threonine-protein kinase at4g08850	49.15	1.29	5.20
BnaC01g09930D	receptor-like protein kinase 5	42.08	2.37	4.73
BnaCnng21280D	g-type lectin s-receptor-like serine threonine-protein kinase rlk1	22.31	2.64	9.25
BnaA04g02560D	probable wrky transcription factor 70	177.15	2.74	30.55
BnaC02g09670D	probable wrky transcription factor 38	45.30	2.58	5.60
BnaA09g07010D	probable wrky transcription factor 51 isoform x1	42.99	1.73	7.54
BnaCnng52600D	probable wrky transcription factor 70	42.77	2.29	6.85
BnaC06g43410D	nac transcription factor 29	42.46	1.83	4.82
BnaC09g06700D	probable wrky transcription factor 51 isoform x2	24.68	-1.59	21.21
BnaC08g27340D	probable wrky transcription factor 70	19.41	1.27	4.84
BnaC05g38130D	nac transcription factor 56-like	17.71	1.29	19.43
BnaC07g49530D	probable wrky transcription factor 50	13.29	2.06	4.49
BnaA07g24270D	nac transcription factor 29	12.47	2.09	4.40

The comparisons were between mock (M) and inoculated (I) samples.

**Table 2:** The primary genes involved in Effector-Triggered Immunity (ETI) based on fold changes in gene regulation associated with the inoculation of three canola lines.

Gene ID	Gene name	15-M vs 15-I	16-M vs 16-I	20-M vs 20-I
BnaA09g44910D	disease resistance protein at4g11170	353.36	4.42	18.54
BnaA03g52640D	probable disease resistance protein	85.56	1.54	5.92
BnaC07g44370D	probable disease resistance protein	23.00	3.74	5.06
BnaC08g15420D	disease resistance protein	22.15	1.60	6.27
BnaA08g24860D	disease resistance protein	16.26	1.18	6.25
BnaA09g47230D	disease resistance protein rfl1-like	13.40	1.74	4.28
BnaA02g16190D	tmv resistance protein n-like	8.98	3.67	18.57
BnaA06g11850D	tmv resistance protein n-like	8.12	2.63	85.02
BnaA09g47300D	disease resistance protein	6.56	2.46	4.24
BnaC06g34080D	tmv resistance protein n-like	5.84	1.20	14.70
BnaA07g30510D	tmv resistance protein n-like	4.6	-1.14	6.40
BnaC01g15190D	tir-nbs-lrr class disease resistance protein	4.34	3.36	4.66
BnaA01g15270D	disease resistance protein rps2	4.10	1.01	4.07
BnaC01g25570D	enhanced disease susceptibility 1	44.86	1.37	13.06
BnaA08g22340D	isochorismate synthase chloroplastic-like	19.34	2.68	20.62
BnaC08g18420D	isochorismate synthase chloroplastic-like	7.14	2.18	8.58

The comparisons were between mock (M) and inoculated (I) samples.

In the MAPK cascade, 95% of 61 associated genes were intensely up-regulated, indicating a strong response caused by the inoculation. The main TF involved in PTI, including probable WRKY TF70, 38 and 51 isoform ×1, as well as NAC TF29, showed the largest fold changes in line 15, followed by line 20, and the smallest or occasionally insignificant changes in line 16 (**Table 1**). Collectively, these results indicated that PTI signaling pathways were activated in lines 15 and 20, but not in line 16.

ETI pathways implicated disease resistance proteins, such as Enhanced Disease Susceptibility 1 (EDS1) and isochorismate synthase, with 13 associated DEGs identified. Their transcription levels were notably up-regulated more in lines 15 and 20 compared to line 16. (**Table 2**; **Supplementary Figure 4B**), including two isochorismate synthases and two isochorismate synthase chloroplastic-like proteins. This would suggest the differential activation of ETI in lines 15 and 20 by pathotype X, but not in line 16. When DEGs involved in abiotic and biotic signaling responses were analyzed for inoculated plants using the MapMan, more MAPK-associated genes, TF and PR genes were up-regulated in lines 15 and 20 than in line 16 (**Supplementary Figures 5**-**7**).

Furthermore, a total of 224 DEGs were identified as common between mock (M)-15/inoculated (I)-15 and M-16/I-16, while only 80 DEGs were shared between M-20/I-20 and M-16/I-16. Additionally, 241 DEGs were shared among M-15/I-15, M-16/I-16 and M-20/I-20. Subsequently, GO analysis was conducted on these shared DEGs (**Supplementary Figures 8**-**10**). DEGs associated with molecular functions were categorized into small molecule/organic cyclic compound binding, transferase, catalytic and hydrolase activities. Biological processes were linked to metabolic processes, cellular responses to stimuli and biological regulation.

### Validation of RNA-seq data quality

**Figure 6:**
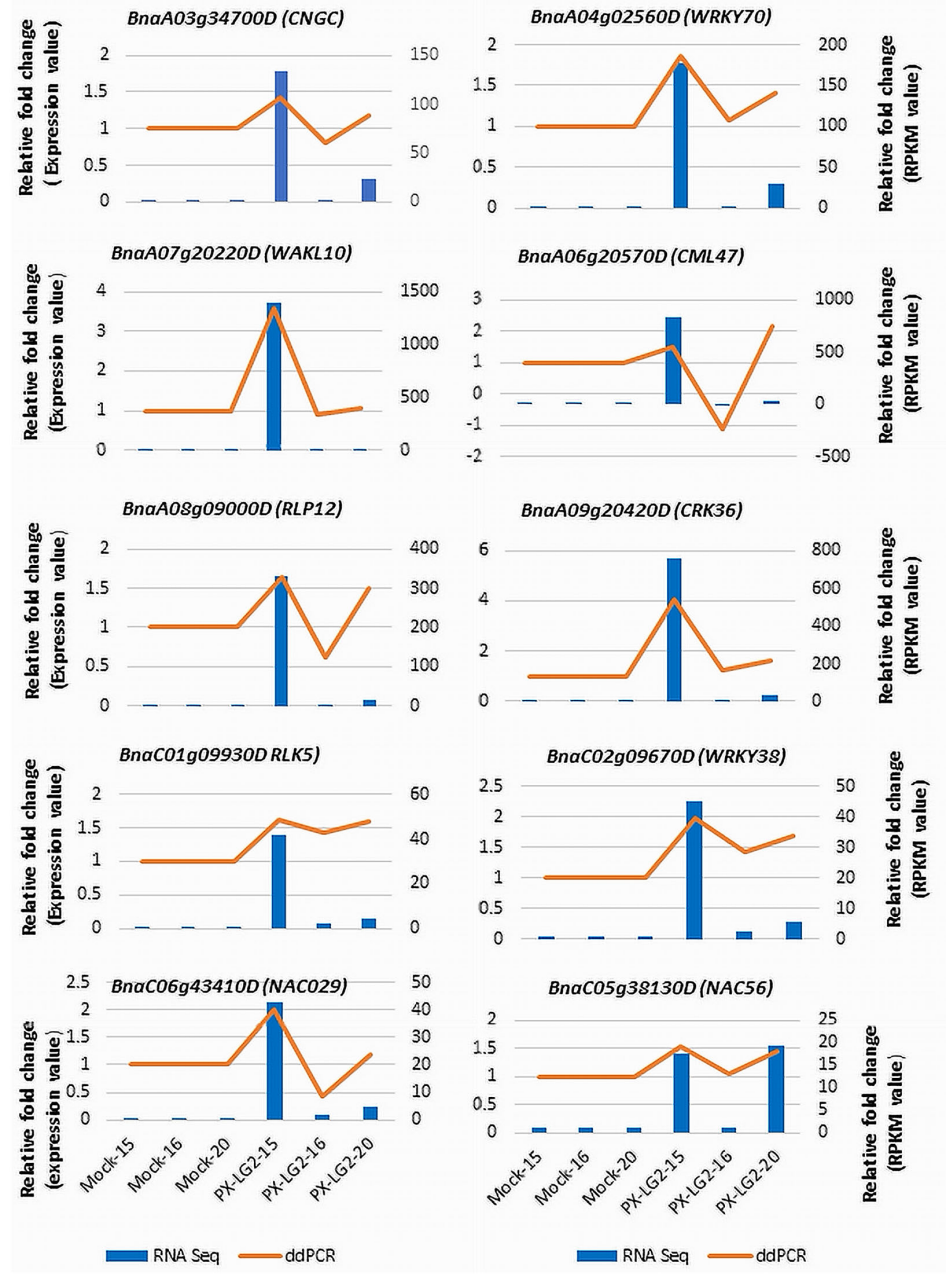
Validation of the RNA-Seq data using ddPCR. The golden line in the chart is intended solely to differentiate the expression of genes in the ddPCR testing from those represented in the RNA-seq data (bar chart). Each ddPCR value is independent, similar to that in the RNA-seq data.

The expression patterns of 10 selected DEGs, involved in pathogen recognition, signal transduction, transcriptional regulation and disease responses, were analyzed using droplet digital PCR (ddPCR). To elucidate the alterations in gene expression following inoculation, relative fold changes in expression levels compared to the mock control were computed based on data obtained from both RNA-Seq and ddPCR (**Figure 6**). The results showed that the expression patterns of the chosen DEGs were similar between ddPCR and RNA-Seq, although the relative fold changes may vary slightly for certain genes between the two methods.

## Discussion

This study used canola lines carrying different CR genes (*CRa^M^* and/or *Crr1^rutb^*) against *P*. *brassicae* pathotype X (L-G2) as a model system to gain insights into molecular clubroot resistance responses. The evaluation of disease severity illustrated that line 16, with the single CR gene *CRa^M^* alone, exhibited susceptibility, while lines 20 and 15, carrying *Crr1^rutb^* alone and *CRa^M^* + *Crr1^rutb^*, respectively, displayed moderate levels of resistance. This observation aligns with previous reports on the effectiveness of certain CR genes and gene combinations in conferring resistance to specific *P*. *brassicae* pathotypes (Strelkov et al., 2018; Sedaghatkish et al., 2019; Tonu et al., 2023).

Stacking the CR genes *CRa^M^* and *Crr1^rutb^* (line 15) provided only partial resistance to the filed population of pathotype X (L-G2) in the current study. It is possible that this partial resistance is derived from *Crr1^rutb^* in line 20, as the inbred line carrying *CRa^M^* alone was susceptible. In a prior study, Tonu et al. (2023) reported that line 20 produced in different batches may vary in resistance to pathotype X, with some batches showing almost immunity. Mapping of *Crr1^rutb^* (Hasan and Rahman, 2016) showed that this CR gene is located in the region in A08 where *Crr1* (Suwabe et al., 2012; Hatakeyama et al., 2013) and *Rcr3* (Karim et al., 2020) also reside. *Rcr3* can be flanked by two SNP markers that are 231.6 Kb apart; this interval contains 32 genes, three of which (Bra020951, Bra020974, Bra020979) are associated with disease resistance. Previous studies also showed that *Crr1* possibly consists of two loci; a major locus, *Crr1a*, which encodes a TIRNB-LRR protein, and a minor locus, *Crr1b* (Suwabe et al., 2012; Hatakeyama et al., 2013). Although it cannot be determined unequivocally whether *Crr1^rutb^* is homologous to *Crr1/Rcr3* due to lacking sequence information on *Crr1^rutb^* and *Rcr3*, it is possible that more than one allele are involved in *Crr1^rutb^* for its strong resistance efficacy against pathotype X (Tonu et al., 2023). It is also possible that one or more of the *Crr1^rutb^* alleles had been lost during the production of lines 15 and 20, as some batches of line 20 showed almost immunity to pathotype X (Tonu et al., 2023). This loss of allele during hybridization has also happened with *Crr1* (Fredua-Agyeman et al., 2018). Despite the partial resistance, line 15, carrying the stacked CR genes *CRa^M^* and *Crr1^rutb^*, showed stronger resistance resilience than line 16 or line 20 in a previous study (Tonu et al., 2023), with little evidence for resistance erosion or soil inoculum buildup over five cycles of continuous exposure to pathotype X.

The RNA-seq analysis provided a comprehensive understanding of the transcriptomic changes in response to the pathotype X (L-G2) inoculation across these three *B. napus* inbred/hybrid lines. The reason for extraction of root-tissue RNA at 14 dpi was that the cortical infection by *P*. *brassicae* would have been completed (McDonald et al., 2014; Irani et al., 2018). Despite the absence of visible symptoms, the expression of genes associated with pathogen recognition, signal transduction and defense responses would have already peaked (Chu et al., 2014). This early time frame is particularly suitable for the RNA-seq analysis, providing valuable insights into plant defense mechanisms against the pathogen attack.

The high-quality sequencing data and subsequent analysis including PCA and ddPCR, ensured the reliability and reproducibility of results. The PCA results indicated tight clustering of biological replicates within each treatment, affirming the consistency of the RNA-seq data. The ddPCR results further supported the transcriptomic data; the generally consistent gene expression patterns observed in both platforms underscored the robustness of DEGs identified. This careful experimental design enhanced the confidence in the transcriptomic changes observed, allowing for meaningful interpretation of the results. The slight differences in gene expression levels (fold changes) between RNA-seq and ddPCR can be due to more sensitive detection of low-abundance transcripts by ddPCR or lower susceptibility of RNA-seq to issues related to PCR efficiency. It is also noteworthy that few distinctions were observed among the DEGs between the control and inoculated line 16 carrying only *CRa^M^*. In contrast, notable disparities were evident between the control and inoculated lines 15 (*CRa^M^* +*Crr1^rutb^*) or line 20 (*Crr1^rutb^*), culminating in a moderately resistant response to L-G2.

The annotation of DEGs revealed substantial transcriptional changes in response to infection by *P*. *brassicae*. The stringent criteria for DEG identification (fold change ≥ 4, FDR ≤ 0.01) ensured the selection of robust candidates. The GO analysis provided insights into the Biological Processes, Molecular Functions, and Cellular Components associated with the DEGs. Notably, the abundance of DEGs involved in biosynthetic processes, response to stress and regulation of cellular processes highlights the complex interplay of molecular events during the plant-pathogen interaction. The comparison of DEG profiles among these three lines shed light on the common and distinct transcriptional responses; lines 15 and 20, both partially resistant to L-G2, shared a substantial number of DEGs, suggesting some common defense mechanisms. Conversely, line 16 exhibited a distinct transcriptional profile, which failed to mount effective resistance.

Plant defense responses involve pathogen recognition, signal transduction and defense activation (Andersen et al., 2018). In the first stage, receptor-like proteins (RLPs) and receptor kinases (RKs) recognize immunogenic molecular patterns of pathogens (Zhou and Zhang, 2020). Up-regulation of several RLPs and RKs, including wall-associated receptor kinase-like 10, cysteine-rich receptor-like protein kinase 36, receptor-like protein 12, and G-type lectin S-receptor-like serine threonine-protein kinase rlk1, was observed in lines 15 and 20, but not in line 16, upon infection (**Table 1**; **Supplementary Figure 4A**). Additionally, the downstream signaling event of pattern recognition receptor (PRR)-based pathogen recognition often involves the mitogen-activated protein kinase (MAPK). Activation of MAPK was strongly evident for lines 15 and 20, with 95% of 61 associated genes being intensely up-regulated and 5% of them down-regulated. In comparison, only 79% of these genes were up-regulated and 21% down-regulated in line 16, with much smaller fold changes (**Supplementary Table 5**).

This upregulation of PRRs, MAPK cascade and TF was generally more pronounced in lines 15 and 20 than in line 16. This strongly indicates the activation of PTI, suggesting robust pathogen recognition and potent PTI-triggered defense responses (Bigeard et al., 2015) against L-G2. In contrast, apparently weaker responses of line 16 in MAPK may suggest a compromised defense mechanism. The high-fold changes of probable WRKY transcription factor would contribute to the activation of PTI responses. In Arabidopsis, activated MAPKs phosphorylate substrate proteins, including ethylene-responsive TF AtERF104 and AtWEKY70 (Bigeard and Hirt, 2018). In the current study, the fold changes for three genes encoding possible WRKY transcription factor 70 were the highest in line 15, lower in line 20 and below the threshold level of significance in line 16 (**Table 1**; **Supplementary Figure 4A**). This emphasizes the strong activation of PTI associated with the double CR genes. There may be complementary interaction between two CR genes (Yu et al., 2022) in response to specific strains of *P. brassicae*, although these enhanced molecular responses did not translate into substantially reduced disease severity (**Figure 1B**).

The analysis of defense responses also revealed the activation of ETI signaling pathways in lines 15 and 20, but not in line 16. The identification of DEGs encoding plant intracellular immune receptors, known as resistance proteins, as well as isochorismate synthases (ICS), supported the notion of ETI. The resistance proteins are believed to trigger ETI upon the detection of pathogen effectors; it appears that EDS1 and ICS are the key components in ETI downstream responses. Up-regulation of EDS1 and two ICS genes was the highest in line 15, followed by line 20. Additionally, DEGs associated with hypersensitive response (HR), characteristic of ETI, were also identified in lines 15 and 20. EDS1 is a general signaling component in the down-stream of NBS-LRR, and is activated by the presence of cleaved products released by resistance proteins (Zhou and Zhang, 2020). EDS1 can combine with phytoalexin deficient 4 (PAD4) or senescence-associated gene 101 to form a complex that induces salicylic acid (SA) biosynthesis or results in cell death (Joglekar et al., 2018). Up-regulation of EDS1 was the highest in line 15 and lowest in line 16 (**Table 2**; **Supplementary Figure 4B**). The high transcription level of EDS1 indicated that it was activated following infection of lines 15 and 20, but not line 16. In Arabidopsis, SA is synthesized by two ICS genes, *ICS1* and *ICS2* (Chen et al., 2009), which were up-regulated in lines 15 and 20 (BnaA08g22340D and BnaC08g18420D), but not in line 16 (**Table 2**; **Supplementary Figure 4B**). In total, thirteen disease resistance-related DEGs involved in ETI were identified, with more pronounced up-regulation in the resistant canola, especially line 15.

A distinguishing characteristic of ETI that sets it apart from PTI is HR (Hatsugai et al., 2017). In our investigation across the three canola lines, a total of 105 genes associated with HR were identified. Notably, 90% of them exhibited up-regulation, while only 10% showed down-regulation in lines 15 and 20. In contrast, line 16 displayed a slightly different pattern, with 80% of the genes up-regulated and 20% down-regulated. However, the fold changes in these genes were markedly higher in lines 15 and 20 compared to line 16 (**Supplementary Table 6**). Collectively, these findings signify the activation of both PTI and ETI for lines 15 and 20 in response to L-G2 inoculation, whereas line 16 failed to exhibit robust activation of these immune responses. Upon the activation of PTI and ETI, the transcriptional reprogramming of plant defense response genes ensued, involving a range of biological processes, including hormone biosynthesis, regulation of SAR and cell wall organization. These processes possibly contributed to the defense mechanism against the L-G2. The recently published study by Wang et al. (2023) identifies WeiTsing (WTS) as a broad-spectrum CR gene inhibiting the colonization of *P. brassicae* in the root stele. Activation of WTS induces the expression of many defense genes associated with plant immunity, notably increasing the levels of RLPs and wall-associated kinases (WAKs). These findings suggest that the activation of WTS by *P. brassicae* likely simultaneously triggers both PTI and ETI signaling pathways, thereby inducing plant immune responses.

## Conclusion

Understanding the molecular mechanisms of CR genes is crucial for efficiently harnessing genetic resources. Single and stacked CR genes offer resistance against specific *P*. *brassicae* pathotypes, enabling the development of breeding strategies with tailored CR profiles. The comparative transcriptome analysis of inbred/hybrid canola lines carrying *CRa^M^*, *Crr1^rutb^*, and *CRa^M^* + *Crr1^rutb^* provides a comprehensive understanding of the host-pathogen interaction involving pathotype X (L-G2). While few distinctions were observed for DEGs in control and inoculated line 16 (*CRa^M^*), noticeable differences were found for lines 20 (*Crr1^rutb^*) and 15 (*CRa^M^* + *Crr1^rutb^*). It appears that the observed resistance may predominantly stem from *Crr1^rutb^* for both lines 15 and 20, given their substantial overlap in DEGs, even though transcription levels were often higher in the double CR-gene line 15. The up-regulation of several RLPs and RKs suggests the activation of PTI during pathogen recognition. Furthermore, activation of ETI is also suggested by the pronounced up-regulation of EDS1, ICS and HR-related genes, especially in line 15. These findings indicated both PTI and ETI contribute to the resistance *CRa^M^* and *Crr1^rutb^* against pathotype X. Upon pathogen recognition, transcriptional reprogramming seems to occur, with several biological processes being activated, including hormone biosynthesis, SAR regulation, and cell-wall organization. This study provides information on intricate molecular interactions involved in CR-mediated resistance and contributes insights for future breeding strategies aimed at sustainable clubroot resistance and management.

## Data availability statement

The original contributions presented in the study are publicly available. This data can be found here: https://www.ncbi.nlm.nih.gov/bioproject/PRJNA1062990.

## Author contributions

RW: Methodology, Writing – original draft, Writing – review & editing, Data curation, Formal Analysis, Investigation, Validation, Visualization. TS: Data curation, Investigation, Methodology, Writing – review & editing, Conceptualization. BG: Investigation, Writing – review & editing. GP: Formal Analysis, Writing – original draft, Writing – review & editing, Conceptualization, Funding acquisition, Methodology, Project administration, Resources, Supervision, Visualization.

## Supplementary material

Supplementary Figure 1 Two-dimensional Principal Component Analysis (PCA) based on the expression of genes across all samples. PC1 is represented on the X-axis, and PC2 on the Y-axis. The proportion of variance for each principal component is denoted in brackets following the axis titles.

Supplementary Figure 2 The top four biological processes of Gene Ontology (GO) assignment associated with up-regulated and down-regulated DEGs from three canola lines.

Supplementary Figure 3 Top twenty GO terms enriched for DEGs in three canola lines.

Supplementary Figure 4 Heatmap for genes involved in PTI and ETI. The comparisons were between mock (M) and inoculated (I) samples. Fold changes were indicated by color scheme, from magenta (high) to green (low) based on their Log-transformed values. Fold-change values are labeled in each cell, with positive and negative values representing up- and down-regulation, respectively. A: Heatmap for PRRs and TF involved in PTI. B: Heatmap of the primary genes involved in ETI.

Supplementary Figure 5 Annotation of DEGs identified in line 15 involving signaling pathways of abiotic/biotic stress using MapMan software. The blue and red colors represent up- and down-regulated DEGs, respectively.

Supplementary Figure 6 Annotation of DEGs identified on line 20 involving signaling pathways of abiotic/biotic stress using MapMan software. The blue and red colors represent up- and down-regulated DEGs, respectively; gray circles represent no DEGs identified in these categories.

Supplementary Figure 7 Annotation of DEGs identified on line 16 involving signaling pathways of abiotic/biotic stress using MapMan software. The blue and red colors represent up- and down-regulated DEGs, respectively.

Supplementary Figure 8 Gene Ontology analysis of DEGs shared between 15-I and 16-I (inoculated). A: DEGs associated with molecular function. B: DEGs linked to biological process.

Supplementary Figure 9 Gene Ontology analysis of DEGs shared between 20-I and 16-I (inoculated). A: DEGs associated with molecular function. B: DEGs linked to biological process.

Supplementary Figure 10 Gene Ontology analysis of DEGs shared among 15-I, 16-I and 20-I (all inoculated). A: DEGs associated with molecular function. B: DEGs linked to biological process.

Supplementary Table 1 Gene information and primer sequences used with ddPCR for validation of RNA-Seq results.

Supplementary Table 2 Summary of RNA-Seq data.

Supplementary Table 3 Summary of DEGs identified in different canola lines.

Supplementary Table 4 DEGs shared between lines 15 and 20, but not by line 16.

Supplementary Table 5 Enrichment analysis of the DEGs involved in the Mitogen-Activated Protein Kinase (MAPK) cascade shared between lines 15 and 20, but not by line 16.

Supplementary Table 6 Enrichment analysis of the DEGs involved in the different biological processes of defense responses.

## References

[B1] Andersen E. J. Ali S. Byamukama E. Yen Y. Nepal M. P. (2018). Disease resistance mechanisms in plants. Genes 9, 339. doi: 10.3390/genes9070339 29973557 PMC6071103

[B2] Balesdent M. H. Gautier A. Plissonneau C. Le Meur L. Loiseau A. Leflon M.. (2022). Twenty years of *Leptosphaeria maculans* population survey in France suggests pyramiding *Rlm3* and *Rlm7* in rapeseed is a risky resistance management strategy. Phytopathol. 112, 2126–2137. doi: 10.1094/PHYTO-04-22-0108-R 35621309

[B3] Bigeard J. Colcombet J. Hirt H. (2015). Signaling mechanisms in pattern-triggered immunity (PTI). Mol. Plant 8, 521–539. doi: 10.1016/j.molp.2014.12.022 25744358

[B4] Bigeard J. Hirt H. (2018). Nuclear signaling of plant MAPKs. Front. Plant Sci. 9, 469. doi: 10.3389/fpls.2018.00469 29696029 PMC5905223

[B5] Brun H. Chevre A. M. Fitt B. D. Powers S. Besnard A. L. Ermel M.. (2010). Quantitative resistance increases the durability of qualitative resistance to *Leptosphaeria maculans* in *Brassica napus* . New Phytol. 185, 285–299. doi: 10.1111/j.1469-8137.2009.03049.x 19814776

[B6] Chen Z. Zheng Z. Huang J. Lai Z. Fan B. (2009). Biosynthesis of salicylic acid in plants. Plant Signal Behav. 4, 493–496. doi: 10.4161/psb.4.6.8392 19816125 PMC2688294

[B7] Chhiba K. D. Hsu C. L. Berdnikovs S. Bryce P. J. (2017). Transcriptional heterogeneity of mast cells and basophils upon activation. J. Immunol. 198, 4868–4878. doi: 10.4049/jimmunol.1601825 28476932 PMC5862545

[B8] Chu M. Song T. Falk K. C. Zhang X. Liu X. Chang A.. (2014). Fine mapping of *Rcr1* and analyses of its effect on transcriptome patterns during infection by *Plasmodiophora brassicae* . BMC Genomics 15, 1–20. doi: 10.1186/1471-2164-15-1166 25532522 PMC4326500

[B9] Conesa A. Götz S. García-Gómez J. M. Terol J. Talón M. Robles M. (2005). Blast2GO: a universal tool for annotation, visualization and analysis in functional genomics research. Bioinformatics 21, 3674–3676. doi: 10.1093/bioinformatics/bti610 16081474

[B10] Diederichsen E. Beckmann J. Schondelmeier J. Dreyer F. (2006). Genetics of clubroot resistance in *Brassica napus*' Mendel'. Acta Hortic. 706, 307–311. doi: 10.17660/ActaHortic.2006.706.35

[B11] Dixon G. R. (2009). The occurrence and economic impact of Plasmodiophora brassicae and clubroot disease. J. Plant Growth Regul. 28, 194–202. doi: 10.1007/s00344-009-9090-y

[B12] Evans C. Hardin J. Stoebel D. M. (2018). Selecting between-sample RNA-Seq normalization methods from the perspective of their assumptions. Brief Bioinform. 19, 776–792. doi: 10.1093/bib/bbx008 28334202 PMC6171491

[B13] Fredua-Agyeman R. Hwang S. F. Strelkov S. E. Zhou Q. X. Feindel D. (2018). Potential of clubroot resistance genes from donor parent *Brassica rapa* subsp. *rapifera* (ECD 04) during doubled haploid production. Plant Pathol. 67, 892–901. doi: 10.1111/ppa.12816

[B14] Fredua-Agyeman R. Rahman H. (2016). Mapping of clubroot disease resistance in spring *Brassica napus* canola introgressed from European winter canola cv. ‘Mendel’. Euphytica 211, 201–213. doi: 10.1007/s10681-016-1730-2

[B15] Gossen B. Strelkov S. Manolii V. Rennie D. Cao T. Hwang S.. (2015). Spread of *Plasmodiophora brassicae* on canola in Canad –2014: old pathogen, new home. Can. J. Plant Pathol. 37, 403–413. doi: 10.1080/07060661.2015.1105871

[B16] Hasan M. J. Rahman H. (2016). Genetics and molecular mapping of resistance to *Plasmodiophora brassicae* pathotypes 2, 3, 5, 6, and 8 in rutabaga (*Brassica napus* var. *napobrassica*). Genome 59, 805–815. doi: 10.1139/gen-2016-0034 27549861

[B17] Hatakeyama K. Suwabe K. Tomita R. N. Kato T. Nunome T. Fukuoka H.. (2013). Identification and characterization of *Crr1a*, a gene for resistance to clubroot disease (*Plasmodiophora brassicae* Woronin) in *Brassica rapa* L. PloS One 8, e54745. doi: 10.1371/journal.pone.0054745 23382954 PMC3559844

[B18] Hatsugai N. Igarashi D. Mase K. Lu Y. Tsuda Y. Chakravarthy S.. (2017). A plant effector-triggered immunity signaling sector is inhibited by pattern-triggered immunity. EMBO J. 36, 2758–2769. doi: 10.15252/embj.201796529 28811287 PMC5599791

[B19] Hirai M. (2006). Genetic analysis of clubroot resistance in. Brassica crops. Breed. Sci. 56, 223–229. doi: 10.1270/jsbbs.56.223

[B20] Hu H. Zhang Z. Yu F. (2024). A CRISPR/Cas9-based vector system enables the fast breeding of selection-marker-free canola with *Rcr1*-rendered clubroot resistance. J. Exp. Bot. 75, 1347–1363. doi: 10.1093/jxb/erad471 37991105 PMC10901203

[B21] Iqbal N. Kumar P. (2022). Integrated COVID-19 Predictor: Differential expression analysis to reveal potential biomarkers and prediction of coronavirus using RNA-Seq profile data. Comput. Biol. Med. 147, 105684. doi: 10.1016/j.compbiomed.2022.105684 35687925 PMC9162937

[B22] Irani S. Trost B. Waldner M. Nayidu N. Tu J. Kusalik A. J.. (2018). Transcriptome analysis of response to *Plasmodiophora brassicae* infection in the Arabidopsis shoot and root. BMC Genomics 19, 1–19. doi: 10.1186/s12864-017-4426-7 29304736 PMC5756429

[B23] Jayavelu N. D. Altman M. C. Benson B. Dufort M. J. Vanderwall E. R. Rich L. M.. (2023). Type 2 inflammation reduces SARS-CoV-2 replication in the airway epithelium in allergic asthma through functional alteration of ciliated epithelial cells. J. Allergy Clin. Immunol. 152, 56–67. doi: 10.1016/j.jaci.2023.03.021 37001649 PMC10052850

[B24] Joglekar S. Suliman M. Bartsch M. Halder V. Maintz J. Bautor J.. (2018). Chemical activation of EDS1/PAD4 signaling leading to pathogen resistance in *Arabidopsis* . Plant Cell Physiol. 59, 1592–1607. doi: 10.1093/pcp/pcy106 29931201

[B25] Karim M. M. Dakouri A. Zhang Y. Chen Q. Peng G. Strelkov S. E.. (2020). Two clubroot-resistance genes, *Rcr3* and *Rcr9^wa^ *, mapped in *Brassica rapa* using bulk segregant RNA sequencing. Int. J. Mol. Sci. 21, 5033. doi: 10.3390/ijms21145033 32708772 PMC7404267

[B26] Kato T. Hatakeyama K. Fukino N. Matsumoto S. (2012). Identificaiton of a clubroot resistance locus conferring resistance to a *Plasmodiophora brassicae* classified into pathotype group 3 in Chinese cabbage (*Brassica rapa* L.). Breed. Sci. 62, 282–287. doi: 10.1270/jsbbs.62.282 23226089 PMC3501946

[B27] Kato T. Hatakeyama K. Fukino N. Matsumoto S. (2013). Fine mapping of the clubroot resistance gene CRb and development of a useful selectable marker in *Brassica rapa* . Breed. Sci. 63, 116–124. doi: 10.1270/jsbbs.63.116 23641188 PMC3621437

[B28] Li Z. K. Sanchez A. Angel E. Singh S. Domingo J. Huang N.. (2001). and Are the dominant and recessive plant disease resistance genes similar? A case study of rice R genes and *Xanthomonas oryzae* pv. *oryzae* races. Genetics 159, 757–765. doi: 10.1093/genetics/159.2.757 11606550 PMC1461810

[B29] Matsumoto E. Ueno H. Aruga D. Sakamoto K. Hayashida N. (2012). Accumulation of three clubroot resistance genes through marker-assisted selection in Chinese cabbage (*Brassica rapa* L. ssp. *pekinensis*). J. Jpn. Soc Hortic. Sci. 81, 184–190. doi: 10.2503/jjshs1.81.184

[B30] Matsumoto E. Yasui C. Ohi M. Tsukada M. (1998). Linkage analysis of RFLP markers for clubroot resistance and pigmentation in Chinese cabbage (*Brassica rapa* ssp. *pekinensis*). Euphytica 104, 79–86. doi: 10.1023/A:1018370418201

[B31] Mcdonald M. Al-Daoud F. Sedaghatkish A. Moran M. Cranmer T. Gossen B. (2020). Changes in the range and virulence of *Plasmodiophora brassicae* across Canada. Can. J. Plant Pathol. 42, 1–7. doi: 10.1080/07060661.2020.1797882

[B32] McDonald M. R. Sharma K. Gossen B. D. Deora A. Feng J. Hwang S. F. (2014). The role of primary and secondary infection in host response to *Plasmodiophora brassicae* . Phytopathol. 104, 1078–1087. doi: 10.1094/PHYTO-07-13-0189-R 24655290

[B33] Mehraj H. Akter A. Miyaji N. Miyazaki J. Shea D. J. Fujimoto R.. (2020). Genetics of clubroot and fusarium wilt disease resistance in *Brassica* vegetables: the application of marker assisted breeding for disease resistance. Plants 9, 726. doi: 10.3390/plants9060726 32526827 PMC7355935

[B34] Orshinsky A. M. Hu J. Opiyo S. O. Reddyvari-Channarayappa V. Mitchell T. K. Boehm M. J. (2012). Correction: RNA-seq analysis of the *sclerotinia homoeocarpa* – creeping bentgrass pathosystem. PloS One 7, 10.1371. doi: 10.1371/annotation/36af97b5-137b-4629-946b-748b63438b03 PMC3414504 22905098

[B35] Peng G. Lahlali R. Hwang S.-F. Pageau D. Hynes R. K. McDonald M. R.. (2014). Crop rotation, cultivar resistance, and fungicides/biofungicides for managing clubroot (*Plasmodiophora brassicae*) on canola. Can. J. Plant Pathol. 36, 99–112. doi: 10.1080/07060661.2013.860398

[B36] Peng G. Pageau D. Strelkov S. E. Gossen B. D. Hwang S.-F. Lahlali R. (2015). A> 2-year crop rotation reduces resting spores of *Plasmodiophora brassicae* in soil and the impact of clubroot on canola. Eur. J. Agron. 70, 78–84. doi: 10.1016/j.eja.2015.07.007

[B37] Rempel C. B. Hutton S. N. Jurke C. J. (2014). Clubroot and the importance of canola in Canada. Can. J. Plant Pathol. 36, 19–26. doi: 10.1080/07060661.2013.864336

[B38] Rosli H. G. Zheng Y. Pombo M. A. Zhong S. Bombarely A. Fei Z.. (2013). Transcriptomics-based screen for genes induced by flagellin and repressed by pathogen effectors identifies a cell wall-associated kinase involved in plant immunity. Genome Biol. 14, R139. doi: 10.1186/gb-2013-14-12-r139 24359686 PMC4053735

[B39] Sedaghatkish A. Gossen B. D. Yu F. Torkamaneh D. McDonald M. (2019). Whole-genome DNA similarity and population structure of *Plasmodiophora brassicae* strains from Canada. R.BMC Gnomics 20, 1–14. doi: 10.1186/s12864-019-6118-y PMC6794840 31619176

[B40] Soneson C. Delorenzi M. (2013). A comparison of methods for differential expression analysis of RNA-seq data. BMC Bioinform. 14, 1–18. doi: 10.1186/1471-2105-14-91 PMC3608160 23497356

[B41] Strelkov S. E. Hwang S. F. (2014). Clubroot in the Canadian canola crop: 10 years into the outbreak. Can. J. Plant Pathol. 36, 27–36. doi: 10.1080/07060661.2013.863807

[B42] Strelkov S. E. Hwang S. F. Manolii V. P. Cao T. Feindel D. (2016). Emergence of new virulence phenotypes of *Plasmodiophora brassicae* on canola (*Brassica napus*) in Alberta, Canada. Eur. J. Plant Pathol. 145, 517–529. doi: 10.1007/s10658-016-0888-8

[B43] Strelkov S. E. Hwang S.-F. Manolii V. P. Cao T. Fredua-Agyeman R. Harding M. W.. (2018). Virulence and pathotype classification of *Plasmodiophora brassicae* populations collected from clubroot resistant canola (*Brassica napus*) in Canada. Can. J. Plant Pathol. 40, 284–298. doi: 10.1080/07060661.2018.1459851

[B44] Suwabe K. Suzuki G. Nunome T. Hatakeyama K. Mukai Y. Fukuoka H.. (2012). Microstructure of a *Brassica rapa* genome segment homoeologous to the resistance gene cluster on Arabidopsis chromosome 4. Breed. Sci. 62, 170–177. doi: 10.1270/jsbbs.62.170 23136528 PMC3405966

[B45] Thimm O. Bläsing O. Gibon Y. Nagel A. Meyer S. Krüger P.. (2004). MAPMAN: a user-driven tool to display genomics data sets onto diagrams of metabolic pathways and other biological processes. Plant J. 37, 914–939. doi: 10.1111/j.1365-313X.2004.02016.x 14996223

[B46] Tonu N. N. Wen R. Song T. Guo X. Murphy L. A. Gossen B. D.. (2023). Canola with stacked genes shows moderate resistance and resilience against a field population of *Plasmodiophora brassicae* (clubroot) pathotype X. Plants 12, 726. doi: 10.3390/plants12040726 36840074 PMC9960129

[B47] Ueno H. Matsumoto E. Aruga D. Kitagawa S. Matsumura H. Hayashida N. (2012). Molecular characterization of the *CRa* gene conferring clubroot resistance in *Brassica rapa* . Plant Mol. Biol. 80, 621–629. doi: 10.1007/s11103-012-9971-5 23054353

[B48] Wang W. Li Q. Zhang W. Tang L. Zhang C. Dong X.. (2023). WeiTsing, a pericycle-expressed ion channel, safeguards the stele to confer clubroot resistance.". Cell 186, 2656–2671. doi: 10.1016/j.cell.2023.05.023 37295403

[B49] Yu F. Zhang Y. Wang J. Chen Q. Karim M. M. Gossen B. D.. (2022). Identification of two major QTLs in *Brassica napus* lines with introgressed clubroot resistance from turnip cultivar ECD01. Front. Plant Sci. 12, 785989. doi: 10.3389/fpls.2021.785989 35095960 PMC8790046

[B50] Zhao S. Ye Z. Stanton R. (2020). Misuse of RPKM or TPM normalization when comparing across samples and sequencing protocols. RNA 26, 903–909. doi: 10.1261/rna.074922.120 32284352 PMC7373998

[B51] Zhou J.-M. Zhang Y. (2020). Plant immunity: Danger perception and signaling. Cell 181, 978–989. doi: 10.1016/j.cell.2020.04.028 32442407

