# Supplementary figures and images for: Comparative transcriptome analysis of canola carrying a single vs stacked resistance genes against clubroot
Source: Front Plant Sci. 2024 May 21;15:1358605. doi: 10.3389/fpls.2024.1358605 (PMC11148231; doi:10.3389/fpls.2024.1358605)

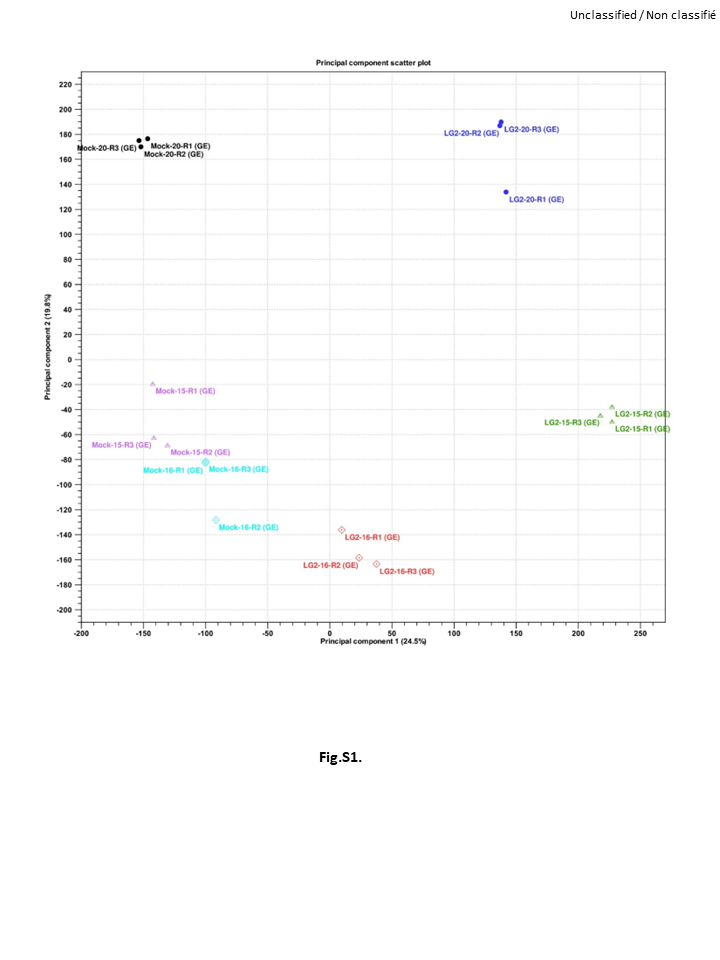

Supplement: Supplementary Figure 1 — Two-dimensional Principal Component Analysis (PCA) based on the expression of genes across all samples. PC1 is represented on the X-axis, and PC2 on the Y-axis. The proportion of variance for each principal component is denoted in brackets following the axis titles. [file Image_1.tif]

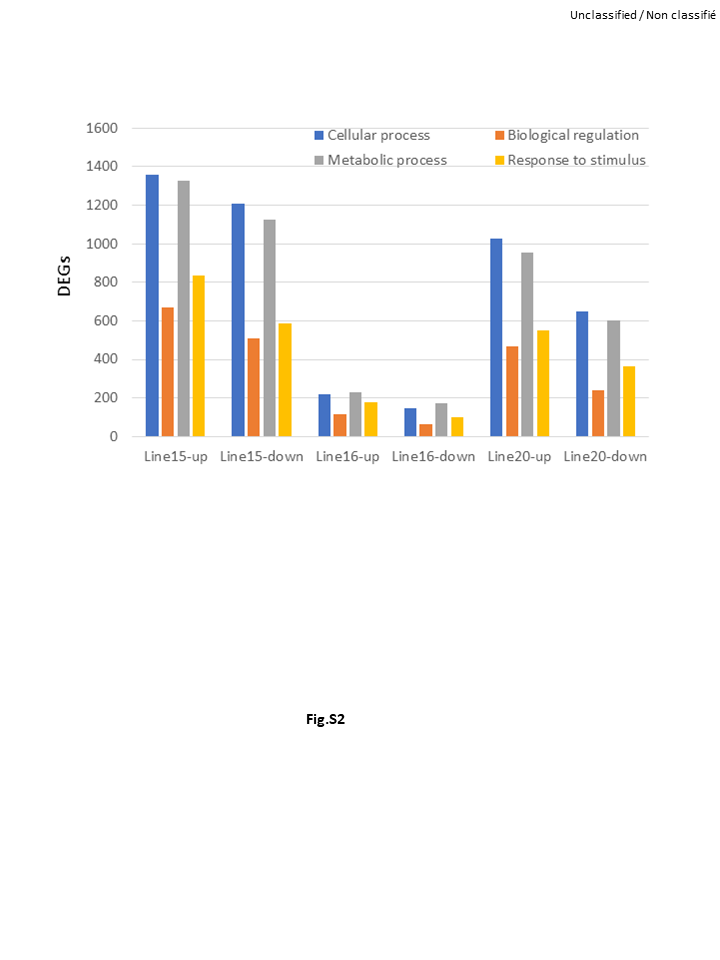

Supplement: Supplementary Figure 2 — The top four biological processes of Gene Ontology (GO) assignment associated with up-regulated and down-regulated DEGs from three canola lines. [file Image_2.tif]

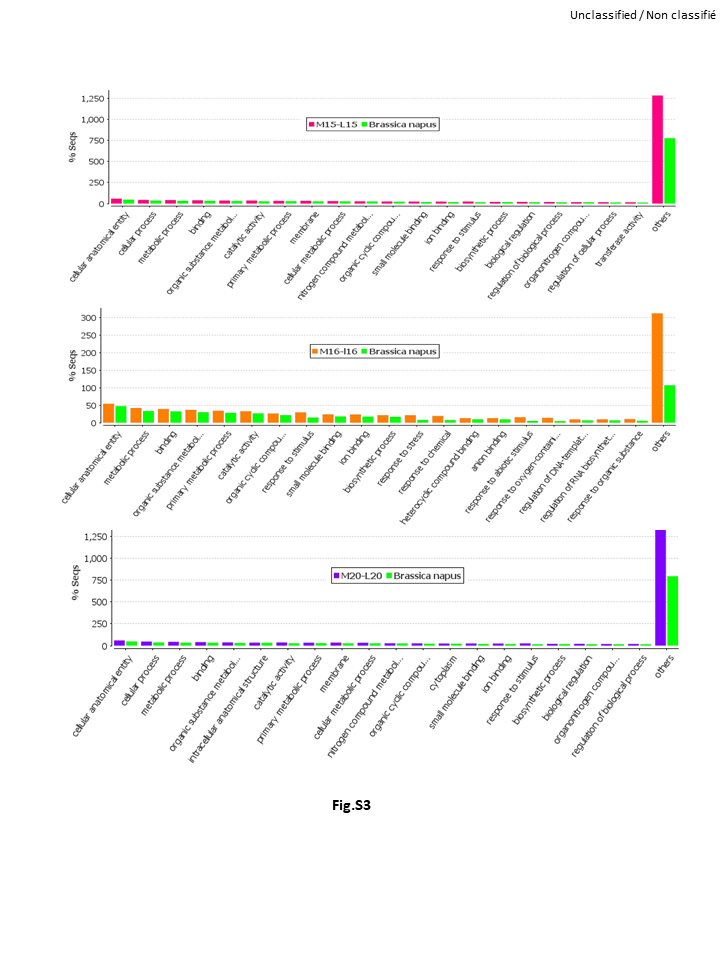

Supplement: Supplementary Figure 3 — Top twenty GO terms enriched for DEGs in three canola lines. [file Image_3.tif]

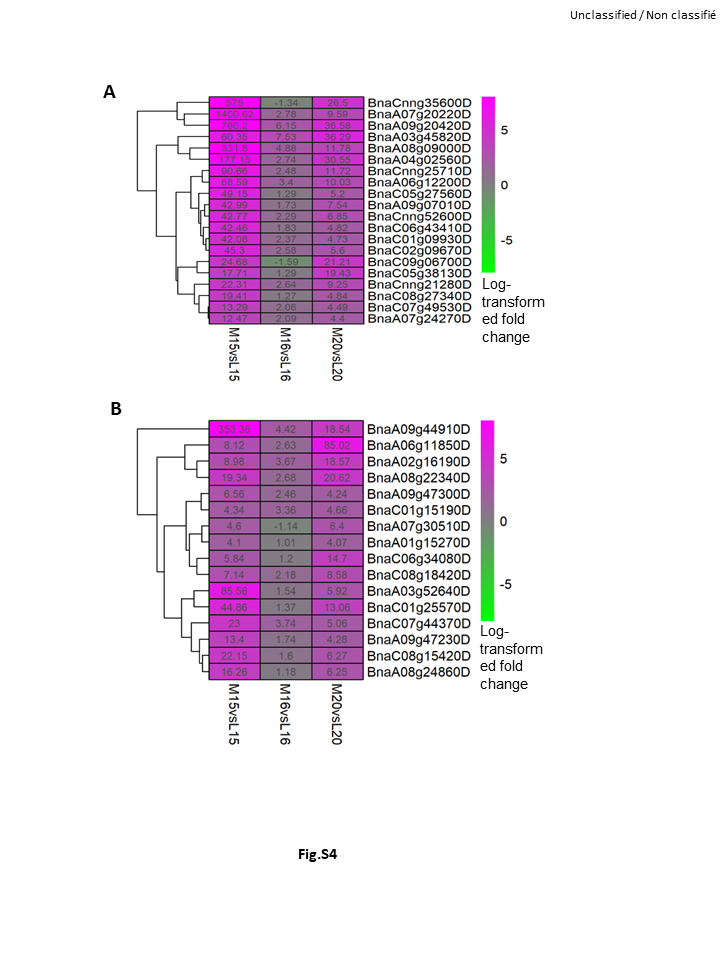

Supplement: Supplementary Figure 4 — Heatmap for genes involved in PTI and ETI. The comparisons were between mock (M) and inoculated (I) samples. Fold changes were indicated by color scheme, from magenta (high) to green (low) based on their Log-transformed values. Fold-change values are labeled in each cell, with positive and negative values representing up- and down-regulation, respectively. A: Heatmap for PRRs and TF involved in PTI. B: Heatmap of the primary genes involved in ETI. [file Image_4.tif]

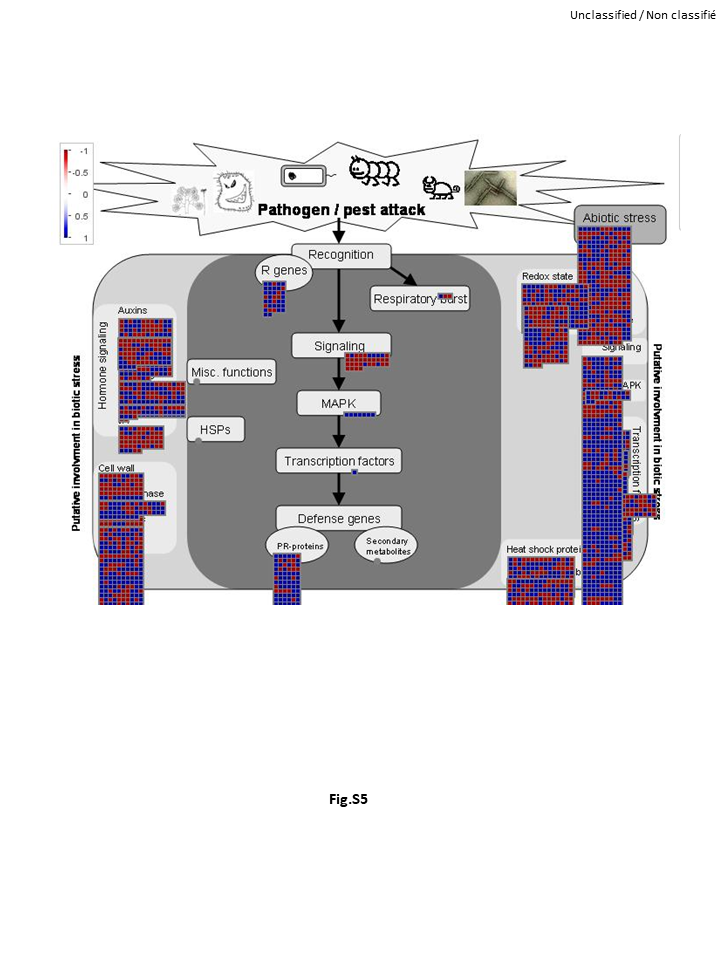

Supplement: Supplementary Figure 5 — Annotation of DEGs identified in line 15 involving signaling pathways of abiotic/biotic stress using MapMan software. The blue and red colors represent up- and down-regulated DEGs, respectively. [file Image_5.tif]

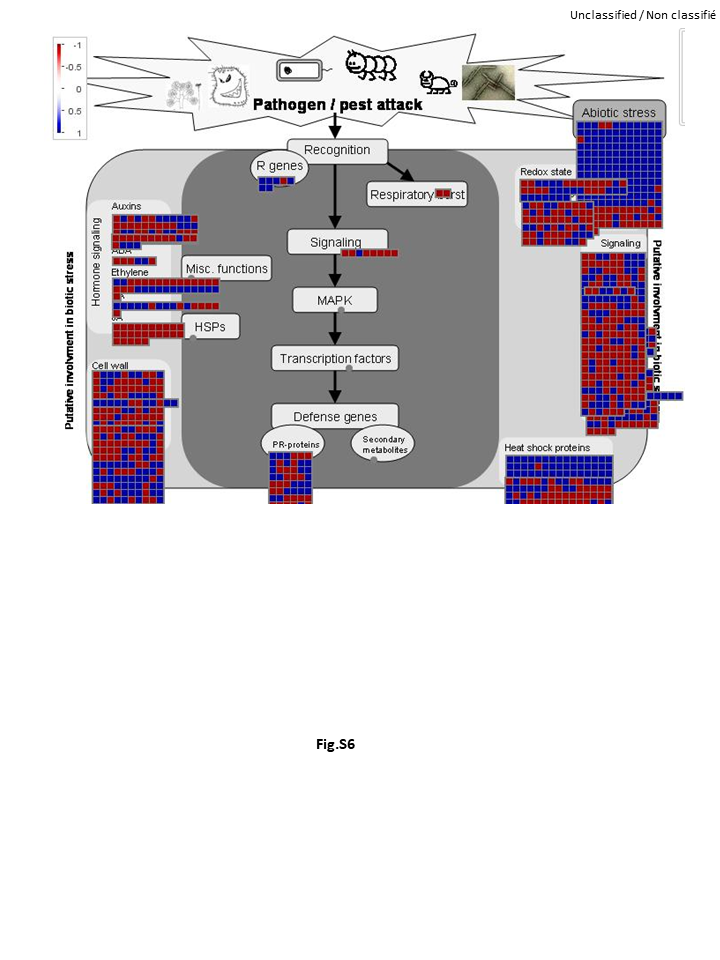

Supplement: Supplementary Figure 6 — Annotation of DEGs identified on line 20 involving signaling pathways of abiotic/biotic stress using MapMan software. The blue and red colors represent up- and down-regulated DEGs, respectively; gray circles represent no DEGs identified in these categories. [file Image_6.tif]

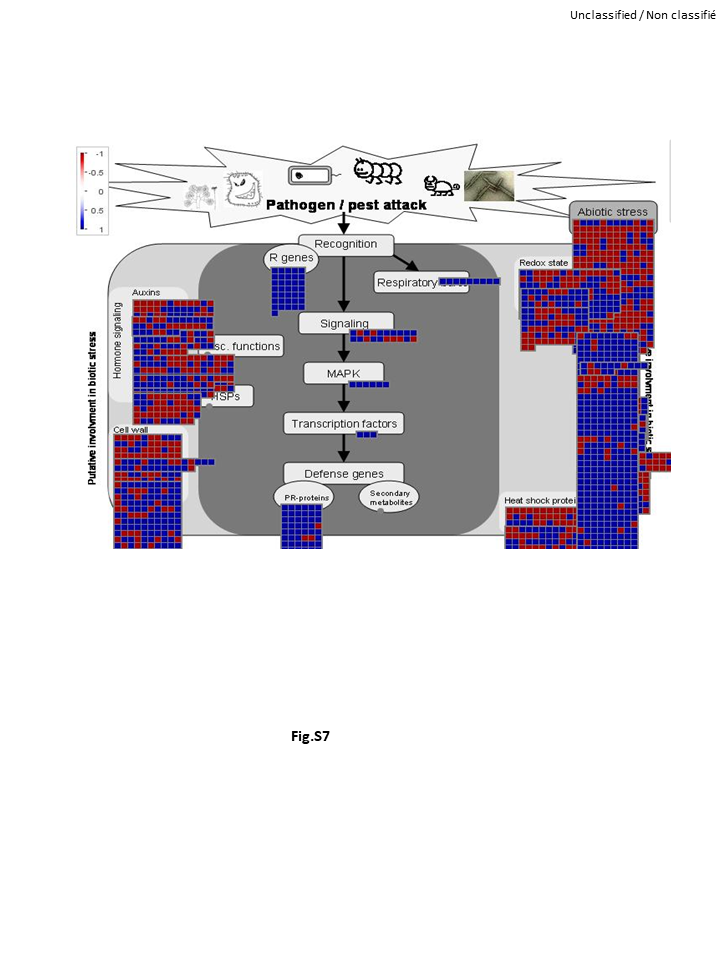

Supplement: Supplementary Figure 7 — Annotation of DEGs identified on line 16 involving signaling pathways of abiotic/biotic stress using MapMan software. The blue and red colors represent up- and down-regulated DEGs, respectively. [file Image_7.tif]

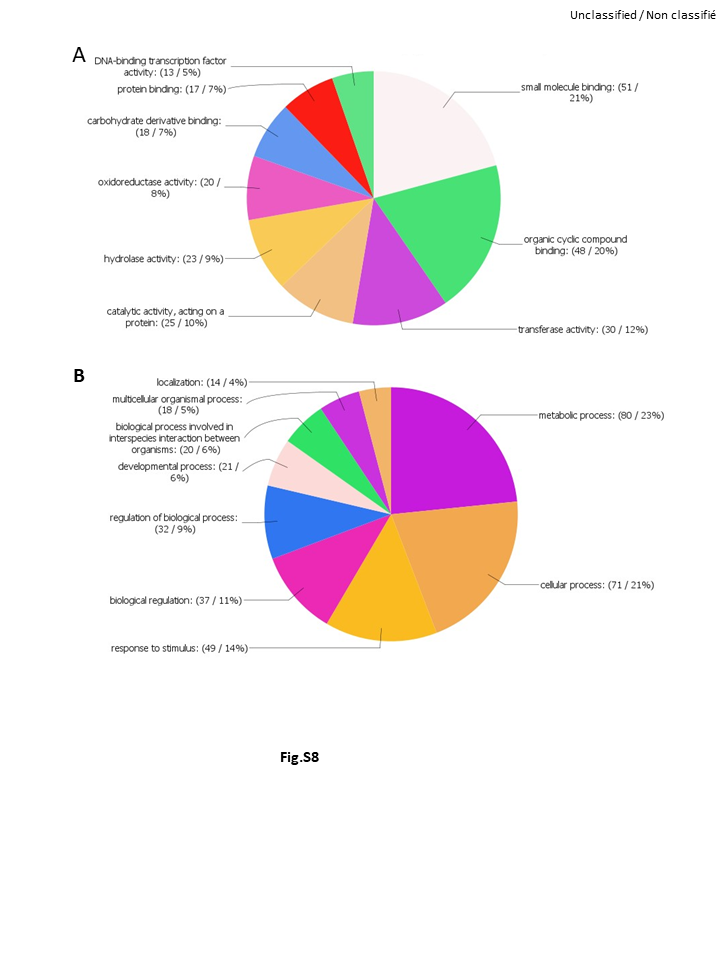

Supplement: Supplementary Figure 8 — Gene Ontology analysis of DEGs shared between 15-I and 16-I (inoculated). A: DEGs associated with molecular function. B: DEGs linked to biological process. [file Image_8.tif]

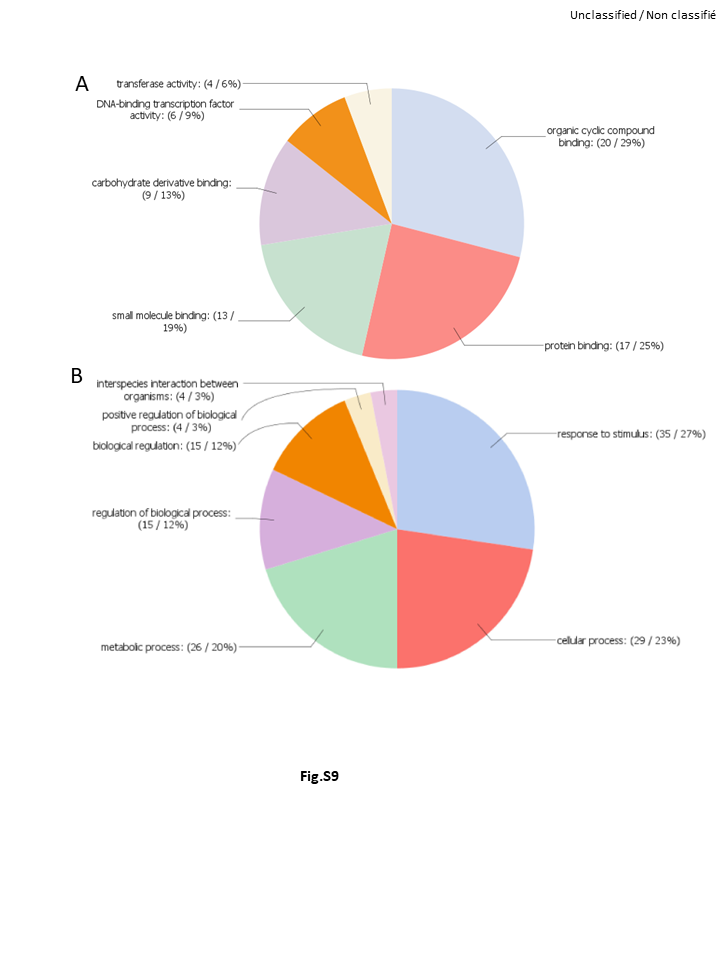

Supplement: Supplementary Figure 9 — Gene Ontology analysis of DEGs shared between 20-I and 16-I (inoculated). A: DEGs associated with molecular function. B: DEGs linked to biological process. [file Image_9.tif]

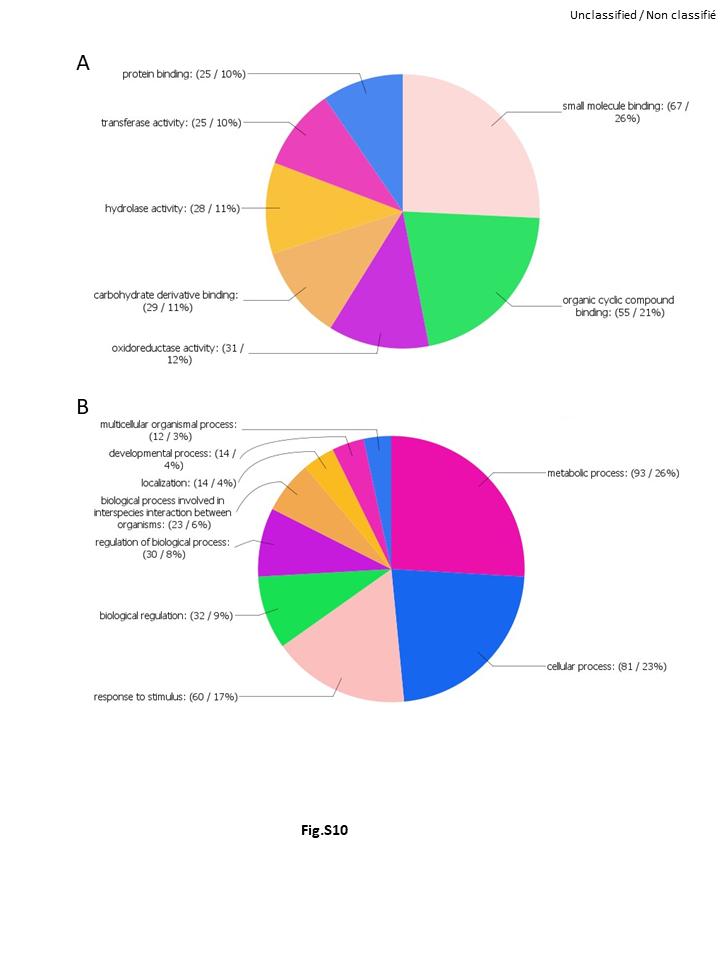

Supplement: Supplementary Figure 10 — Gene Ontology analysis of DEGs shared among 15-I, 16-I and 20-I (all inoculated). A: DEGs associated with molecular function. B: DEGs linked to biological process. [file Image_10.tif]
